# Association of triglyceride glucose-body mass index (TyG-BMI) with metabolic dysfunction-associated steatotic liver disease: A systematic review and meta-analysis

**DOI:** 10.1371/journal.pone.0324483

**Published:** 2025-08-04

**Authors:** Yasaman Ghodsi Boushehri, Zahra Meymanatabadi, Ali Ezzatollahi Tanha, Pouria Azami, Maryam Alaei, Amir Ali Alamdari, Hooman Momtazi, Nasrin Deilami Moezzi, Amirhossein Habibzadeh, Shaghayegh Khanmohammadi

**Affiliations:** 1 School of Medicine, Shiraz University of Medical Sciences, Shiraz, Iran; 2 School of Medicine, Qazvin University of Medical Sciences, Qazvin, Iran; 3 School of Medicine, Tehran University of Medical Sciences, Tehran, Iran; 4 School of Medicine, Shahid Beheshti University of Medical Sciences, Tehran, Iran; 5 School of Dentistry, Islamic Azad University of Medical Sciences, Tehran, Iran; 6 Department of Clinical Biochemistry, School of Pharmacy & Pharmaceutical Sciences, Isfahan University of Medical Sciences, Isfahan, Iran; 7 Non-Communicable Diseases Research Center, Endocrinology and Metabolism Population Sciences Institute, Tehran University of Medical Sciences, Tehran, Iran; 8 Research Center for Immunodeficiencies, Pediatrics Center of Excellence, Children’s Medical Center, Tehran University of Medical Sciences, Tehran, Iran; Universita degli Studi della Campania Luigi Vanvitelli Scuola di Medicina e Chirurgia, ITALY

## Abstract

**Background:**

TyG-BMI has been proposed as a marker of insulin resistance in metabolic-associated fatty liver disease, but its clinical utility remains uncertain. This study aims to evaluate the association between TyG-BMI and metabolic dysfunction-associated steatotic liver disease (MASLD) through a systematic review and meta-analysis, focusing on the diagnostic performance across different subgroups.

**Methods:**

A comprehensive literature search was conducted in PubMed, Scopus, Embase, and Web of Science up to January 20, 2025. Studies evaluating the relationship between TyG-BMI and MASLD in adults were included. A random-effects model was employed to pool effect sizes, and subgroup analyses were conducted based on sex, disease definition, and population type.

**Results:**

Thirty-five studies with 339,087 participants were included. The pooled mean difference for TyG-BMI between MASLD and non-MASLD groups was 42.72 (95% CI: 35.93–49.51; p < 0.0001). Subgroup analysis revealed higher mean differences in the metabolic-associated fatty liver disease (MAFLD) group (49.56, 95% CI: 39.38–59.74) compared to non-alcoholic fatty liver disease ase (NAFLD) (34.68, 95% CI: 28.45–40.91). The odds ratio per one-unit increment of the TyG-BMI was 1.05 (95% CI: 1.03–1.08). Sensitivity for TyG-BMI in diagnosing MASLD was 0.79 (95% CI: 0.73–0.84), and specificity was 0.76 (95% CI: 0.71–0.80). The pooled area under the curve (AUC) for TyG-BMI was 0.83 (95% CI: 0.81–0.86), with better performance in females (0.88) compared to males (0.83). Subgroup analysis by disease definition showed a higher AUC for MAFLD (0.87) compared to NAFLD (0.81).

**Conclusion:**

TyG-BMI is a promising diagnostic marker for MASLD, with higher diagnostic performance in MAFLD and among females. Further studies are needed to confirm these findings in diverse populations.

## Introduction

Metabolic dysfunction-associated steatotic liver disease (MASLD), formerly known as non-alcoholic fatty liver disease (NAFLD), is one of the most common causes of chronic liver disease worldwide, with an increasing prevalence due to the rising global burden of metabolic disorders such as obesity, type 2 diabetes mellitus (T2DM), and hypertension [1]. In comparison to NAFLD, MASLD is characterized by excessive fat accumulation in the liver, independent of significant alcohol consumption, and is associated with various metabolic abnormalities, including insulin resistance, dyslipidemia, and hypertension. As MASLD progresses, it may lead to severe complications, such as liver cirrhosis, hepatocellular carcinoma, and cardiovascular disease, making early diagnosis and intervention essential [2,3].

The TyG index, calculated as the product of fasting triglycerides and fasting glucose, has been recognized as a reliable surrogate marker for insulin resistance. Research indicates that the TyG index alone is a useful tool for identifying individuals at risk for T2DM, cardiovascular diseases, and metabolic syndrome [4]. The TyG index was recently shown to be a useful tool for identifying patients with MASLD, especially in lean patients [5,6]. Recently, other TyG index-related surrogate markers, such as TyG-waist circumference (WC), and TyG-waist circumference-to-height ratio (WHtR) have emerged. The triglyceride glucose-body mass index (TyG-BMI) is an emerging, simple, and cost-effective biomarker that combines triglyceride levels, glucose levels, and body mass index (BMI). TyG-BMI has been proposed as a surrogate marker for insulin resistance and a predictor of various disorders, including MASLD, cardiovascular diseases, and T2DM [7,8]. When BMI is included in the calculation, the resulting TyG-BMI index provides a more nuanced understanding of metabolic health, particularly in populations with varying body compositions. Studies have shown that TyG-BMI outperforms the TyG index in predicting insulin resistance, as it accounts for body fat distribution and obesity’s role in metabolic health [9].

Several studies have investigated the association between TyG-BMI and MASLD, but the results remain inconsistent, particularly when considering differences by sex and across various study groups [10–12]. This inconsistency highlights the need for further research to address these discrepancies and provide a more comprehensive understanding of the relationship between TyG-BMI and MASLD. Given the clinical importance of identifying reliable biomarkers for early detection and risk stratification of MASLD, a comprehensive evaluation of the available evidence is needed.

This systematic review and meta-analysis aim to synthesize the existing literature on the association between TyG-BMI and MASLD. By pooling data from multiple studies, we seek to provide a clearer understanding of the potential role of TyG-BMI as a diagnostic and prognostic tool in MASLD and to explore the strength and consistency of this association across different populations and study designs. We have used the latest term, MASLD, throughout the manuscript. However, all fatty liver diseases defined by NAFLD, MAFLD, and MASLD were considered in this study. Also, due to the limited number of studies using MASLD definition, we merged MASLD and MAFLD patients for subgroup analysis by disease definition.

## Materials and methods

### Study design

This systematic review and meta-analysis aimed to investigate the association between the TyG-BMI index and MASLD. The review was conducted following the Preferred Reporting Items for Systematic Reviews and Meta-Analyses (PRISMA) guidelines (Registration number: CRD42024554538).

### Literature search

We conducted a comprehensive literature search in the following electronic databases: PubMed, Scopus, Embase, and Web of Science. The search strategy was developed using a combination of MeSH terms and keywords related to “TyG-BMI,” “NAFLD,” “MASLD, “ and “MAFLD.” Studies were investigated from inception up to January 20, 2025. We also reviewed the reference lists of included studies and relevant reviews to identify additional articles. The full version of the search strategy is available in S1 Table.

### Eligibility criteria

We included observational studies that assessed the relationship between TyG-BMI MASLD in adult populations. Studies meeting the following criteria with no time and language restrictions were eligible: (1) adults aged 18 years or older; (2) studies that measured TyG-BMI (formula: TyG-BMI = (ln(fasting triglycerides (mg/dL)×fasting glucose (mg/dL))/2)×BMI) and evaluated its association and predictability power in diagnosing MASLD; (3) observational studies, including cross-sectional, cohort, and case-control studies. Studies were excluded if they: focused on animal models or children, used non-validated diagnostic criteria for MASLD, and did not report sufficient data to calculate effect sizes or outcomes related to TyG-BMI. Letters, commentaries, reviews, case reports, and case series were also deemed ineligible and, therefore, excluded from the systematic review.

After removing duplicates, two authors (YGB and ZM), independently, assessed the titles and abstracts of all identified papers to determine eligibility according to the predefined inclusion and exclusion criteria. Subsequently, both authors independently conducted a thorough review of the full texts of studies that met these criteria. Any disagreements during the review process were resolved through consensus.

### Data extraction

Two independent reviewers (YGB and AET) extracted the following data from each eligible study including, study characteristics (first author, year of publication, study design, sample size, country of origin, follow-up duration), population characteristics (age, sex, and comorbid conditions), and outcome data (MASLD diagnosis criteria, effect estimates (odds ratios [OR], hazard ratios [HR], mean/median TyG-BMI in each group, sensitivity, specificity, area under the curve [AUC]), and their 95% confidence intervals [CIs] and standard deviation [SD]). Discrepancies between the two reviewers were resolved through discussion or by consulting a third reviewer (MA).

### Risk of bias assessment

The methodological quality and risk of bias of included studies were assessed using the Newcastle-Ottawa Scale (NOS) for cohort, cross-sectional, and case-control studies. Two independent authors (AH and PA) assessed the qualities and in case of disagreement, a third author (AAA) resolved the issue. Studies scoring 7–9 stars were considered high quality, indicating a low risk of bias. Those scoring 4–6 stars were deemed to be of fair quality, suggesting a moderate risk of bias. Studies scoring 0–3 stars were classified as poor quality, indicating a high risk of bias.

### Statistical analysis

The statistical analysis for this meta-analysis was performed using R version 4.4.2 with the ‘meta’ and ‘metafor’ packages [13]. The primary outcomes of interest included the AUC, sensitivity, specificity, ORs, and mean differences of the TyG-BMI in association with MASLD. For each included study, we extracted AUCs, ORs, sensitivity, specificity, and TyG-BMI mean in each study group, along with their respective CIs or SDs. Among studies using the same database (e.g., NHANES), we included the most general population with the highest sample size. The methods proposed by Wan et al. [14] and Luo et al. [15] were used to convert median and interquartile range values into means and SDs. In cases where the 95% CI for the AUC was not provided, we applied the Hanley and McNeil (1982) [16] method to calculate it. A random-effects model was employed to pool the data, accounting for both within-study and between-study variability. Specifically, we used the restricted maximum likelihood (REML) method to estimate the between-study variance (τ²), which is particularly suitable given the expected heterogeneity among the studies. Heterogeneity was assessed using the I² statistic and Cochran’s Q test. A p-value of < 0.05 was considered statistically significant. An I² value greater than 50% or a significant Q test (p < 0.10) was considered indicative of substantial heterogeneity. Subgroup analyses were performed based on sex, disease definition, and population type where applicable. Also, due to the limited number of studies using MASLD definition, we merged MASLD and MAFLD patients for subgroup analysis by disease definition. Publication bias was assessed using Begg’s [17] and Egger’s tests [18] and visual inspection of funnel plots. Asymmetry in the funnel plots or a significant Egger’s test (p < 0.05) was considered indicative of potential publication bias.

## Result

### Study selection

**Fig 1:**
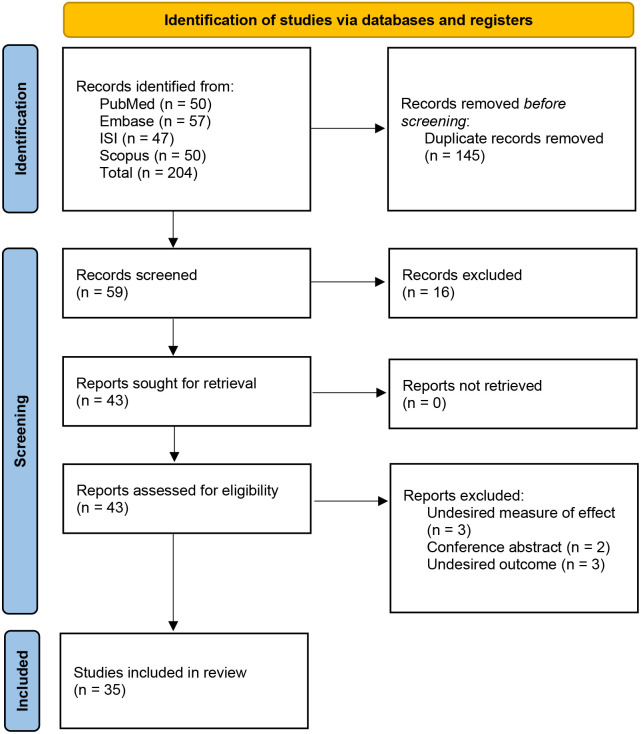
PRISMA flowchart.

The initial search identified a total of 204 results: 57 from Embase, 47 from ISI, 50 from PubMed, and 50 from Scopus (S2 Table). After merging the results and removing duplicates, 59 unique studies remained. These 59 studies were screened for eligibility based on titles and abstracts, resulting in the selection of 43 studies for full-text review. Following a thorough review of the full texts, eight studies were excluded (3 due to an undesired measure of effect, 2 as conference abstracts, and 3 due to undesired outcomes) (S3 Table). As a result, 35 studies were deemed eligible and included in the qualitative synthesis for the meta-analysis. Fig 1 presents a flow diagram of the study selection process.

### Basic characteristics

Overall, 35 studies with a total number of 339,087 individuals from countries including China (n = 23), Korea (n = 4), Iran (n = 3), Japan (n = 2), Tanzania (n = 1), Mexico (n = 1), Ghana (n = 1). Of the included studies, 33 had a cross-sectional design, one was a cohort study, and one was a case-control study. Four studies used the NHANES database, and two studies used the ‘DATADRYAD’ database. Among the included studies, 24 focused on the general population, 5 on individuals with T2DM, 4 on normal BMI individuals, and 2 on overweight individuals and patients with obesity. Ultrasound, transient elastography, and CT-scan were among the imaging techniques to diagnose fatty liver disease. The basic characteristics of the included studies and effect sizes are summarized in Tables 1 and 2.

**Table 1:** Basic characteristics of the included studies.

^Author, year^	^Country^	^Study design^	^Population^	^Sample size (F)^	^NAFLD N^	^non-NAFLD N^	^M/NAFLD diagnosis^	^Age range^	^Mean/Median age^	^Adjustments^
^Bockarie, 2023^ [10]	^Ghana^	^Cross-sectional^	^General population^	^210 (152)^	^52^	^158^	^Ultrasound^	^≥18 years^	^54 (IQR 42–61)^	^Age, sex, smoking, alcohol consumption, SBP, DBP^
^Chang, 2023^ [11]	^China^	^Cross-sectional^	^General population^	^20922 (8579)^	^8099^	^12823^	^Ultrasound+MAFLD criteria^	^18-80^	^MAFLD:46.91 (12.57); non-MAFLD: 42.16 (12.65)^	^Age, sex, blood pressure, fasting glucose, blood lipids and liver and kidney function^
					^M:6152^	^M:6191^				
^Chen, 2024^ [19]	^China^	^Case-control^	^MAFLD and control^	^2361 (985)^	^649^	^1712^	^Ultrasound+MAFLD criteria^	^≥18 years^	^NR^	^Sex, age, education level, marital status, income, smoking status, alcohol drink status, tea drink status, physical activity, BMI, SBP, DBP, TG, TC, HDL-C, LDL-C, ALT, AST, GGT, FPG.^
^Gao, 2022^ [20]	^China^	^Cross-sectional^	^T2DM^	^190 (102)^	^34^	^156^	^Liver biopsy, imaging, and blood biomarkers+MAFLD criteria^	^≥18 years^	^MAFLD: 50 (IQR 46–60); non-MAFLD: 57 (IQR 46–64)^	^Sex, drinking history, duration of diabetes, TG, ALP, AST, and BUA^
^Han, 2024^ [21]	^Korea^	^Cross-sectional^	^General population^	^852 (343)^	^150^	^702^	^CT scan+MAFLD criteria^	^≥19 years^	^non-MAFLD: 52.77 (9.96); MAFLD: 51.86 (9.72)^	^NA^
					^M:128^	^M:381^				
^Hu, 2022^ [22]	^Japan^	^Cross-sectional^	^General population (DATADRYAD’ database)^	^7118 (3394)^	^1272^	^5846^	^Ultrasound^	^≥18 years^	^43.533 (8.918)^	^NA^
					^M: 1031^	^M: 2693^				
^Hu, 2025^ [8]	^China^	^Cross-sectional^	^T2DM^	^1742 (824)^	^996^	^746^	^Ultrasound+T2DM^	^NR^	^non-MASLD: 60(52.5–67.5); MASLD: 54(42–66)^	^Gender, age, smoking status, drinking status and education levels^
^Khamseh, 2021^ [23]	^Iran^	^Cross-sectional^	^Obesity/overweight^	^184 (91)^	^96^	^88^	^Transient elastography^	^30-65^	^non-NAFLD: 45.0 (8.7); NAFLD: 44.4 (9.3)^	^Age, gender, waist-to-hip ratio, SBP, DBP, serum cholesterol, ALT, AST, HOMA-IR, statin medication, smoking and physical activity^
^Khamesh, 2024^ [24]	^Iran^	^Cross-sectional^	^General population^	^644 (342)^	^320^	^324^	^Transient elastography+MAFLD criteria^	^25-75^	^non-MAFLD: 49.8(9.8); MAFLD: 50.7 (9.1)^	^NA^
^Kilonzo, 2024^ [12]	^Tanzania^	^Cross-sectional^	^Obesity/overweight^	^181 (127)^	^55^	^126^	^Ultrasound^	^≥18 years^	^NR^	^NR^
^Kim, 2023^ [25]	^Korea^	^Cross-sectional^	^General population^	^22391 (11044)^	^8246^	^14145^	^Ultrasound+MAFLD criteria^	^NR^	^non-MAFLD: 47.9 (12.9); MAFLD: 51.50 (11.5)^	^Age, gender, AST, ALT, GGT, SBP, DBP, HTN, DM, DL, smoking, and exercise^
					^M:5737^	^M:5610^				
^Kim, 2021^ [26]	^Korea^	^Cross-sectional^	^General population^	^10585^	^3284^	^7301^	^Ultrasound^	^NR^	^48.1 (8.7)^	^Age, sex, alcohol, exercise, smoking, SBP, TC, hsCRP, HbA1c, AST, ALT, and GGT^
^Lee, 2024^ [27]	^Korea^	^Cross-sectional^	^General population^	^1382 (27)^	^901^	^481^	^Transient elastography+MASLD criteria^	^18-35^	^23.3(5.0)^	^NA^
^Li D., 2024^ [28]	^China^	^Cross-sectional^	^General population^	^8401 (3680)^	^2162^	^6239^	^Ultrasound^	^NR^	^40 (15)^	^Age, sex, smoking, ALT, AST, and TC^
					^M: 1725^	^M: 2996^				
^Li H., 2023^ [29]	^China^	^Cross-sectional^	^General population^	^72225 (37937)^	^18347^	^53878^	^Ultrasound^	^≥60 years^	^69.93 (6.72)^	^Age, sex, marital status, smoking, physical exercise, SBP, RHR^
^Li N., 2022^ [30]	^China^	^Cross-sectional^	^T2DM^	^826 (273)^	^552^	^274^	^Ultrasound^	^NR^	^NAFLD:55 (47–64); non-NAFLD: 59 (49–67)^	^Age, gender, BMI, SBP, DBP, diabetes duration, fasting and postprandial blood glucose^
					^M:375^	^M: 178^				
^Li S., 2023^ [31]	^China (US population)^	^Cross-sectional^	^Non-obese^	^1776 (909)^	^784^	^992^	^Transient elastography^	^≥20 years^	^48.6 (95%CI:46.9–50.3)^	^Age, BMI, gender, ethnic, education level, alcohol use, diabetes, HTN, physical activity and TC^
^Li Y., 2020^ [32]	^China^	^Cohort (5 years follow-up)^	^Non-obese^	^9767^	^841^	^8926^	^Ultrasound^	^14-90^	^42.5 (14.7)^	^Sex, age, ALP, GGT, ALT, AST, ALB, GLB, Cr, UA, FPG, TG, HDL-C, LDL-C, SBP, DBP, DBIL^
^Liu, 2022^ [33]	^China^	^Cross-sectional^	^General population^	^229^	^97^	^132^	^Ultrasound+MAFLD criteria^	^NR^	^non-MAFLD: 38.93(9.51); MAFLD: 42.59 (12.64)^	^Age, gender, and BMI^
					^M: 75^	^M: 80^				
^Malek, 2021^ [34]	^Iran^	^Cross-sectional^	^T2DM^	^175 (95)^	^122^	^53^	^Transient elastography^	^30-65^	^non-NAFLD:46.72(8.53); NAFLD: 49.89(8.65)^	^Age, gender, waist to hip ratio, SBP, diastolic blood pressure, serum cholesterol, HDL, LDL, ALT, AST, A1C, HOMA-IR, statin medication, diabetes duration, smoking, and physical activity^
^Otsubo, 2023^ [35]	^Japan^	^Cross-sectional^	^Non-obese^	^24825 (11523)^	^4352^	^20473^	^Ultrasound^	^≥20 years^	^44.3(10.0)^	^Age, sex, HbA1c, AST, ALT, HDL, LDL, UA, eGFR, WBC, current smoker, never drinker, training, sleeping disorder^
					^M: 3619^	^M: 9683^				
^Peng, 2023^ [36]	^China (US population)^	^Cross-sectional^	^General population (NHANES)^	^MAFLD: 809 (401)^	^478^	^331^	^Ultrasound+MAFLD criteria^	^≥ 20 years^	^non-MAFLD: 40.00 (28.50–57.00); MAFLD: 50.00 (37.00–61.00)^	^Independent variables, gender, age, ethnicity, FIPR, education level, disease history (HTN, high cholesterol and diabetes)^
				^NAFLD: 809 (401)^	^499^	^310^	^Ultrasound^		^non-NAFLD:41.00 (29.25–57.00); NAFLD: 49.00 (36.00–61.00)^	
					^MAFLD M: 251^	^MAFLD M: 157^				
					^NAFLD M:267^	^NAFLD M:141^				
^Priego-Parra, 2024^ [37]	^Mexico^	^Cross-sectional^	^General population^	^161 (99)^	^122^	^39^	^Transient elastography+MASLD criteria^	^≥18 years^	^non-MASLD:52 (45–64); MASLD: 56 (47–64)^	^NA^
					^M: 53^	^M: 9^				
^Sheng, 2021^ [38]	^China^	^Cross-sectional^	^General population^	^14251 (6870)^	^2507^	^11744^	^Ultrasound^	^NR^	^F: 43.27; M: 44.78^	^Age, habit of exercise, GGT, TC, HDL-C, HbA1c, smoking status, drinking status and DBP^
					^M: 2029^	^M:5382^				
^Song, 2022^ [39]	^Korea^	^Cross-sectional^	^General population^	^17577 (9908)^	^6856^	^10721^	^Ultrasound^	^≥ 20 years^	^NAFLD:51.1(11.8); non-NAFLD: 47.5 (13.2)^	^Age, AST, ALT, SBP, DBP, smoking, exercise, and type 2 diabetes^
					^M: 4127^	^M: 3542^				
^Tian, 2024^ [40]	^China^	^Cross-sectional^	^T2DM^	^268 (104)^	^145^	^123^	^Transient elastography^	^>18^	^NAFLD: 49.0 (40.0–60.0); non-NAFLD: 56.0 (47.0–61.0)^	^Age, sex, blood pressure, BMI, fasting glucose, blood lipids and liver and kidney function^
					^M: 93^	^M: 71^				
^Wang M., 2023^ [41]	^China^	^Cross-sectional^	^General population^	^1171 (228)^	^737^	^434^	^Transient elastography^	^≥18 years^	^non-NAFLD (M): 48.75 (12.29); NAFLD: 47.81 (11.56); non-NAFLD (F): 44.82 (12.50); NAFLD: 50.51 (11.29)^	^NR^
					^M:637^	^M:306^				
^Wang R., 2021^ [42]	^China^	^Cross-sectional^	^General population^	^14251 (6840)^	^2507^	^11744^	^Ultrasound^	^NR^	^43.53^	^Sex, age, ALT, AST, habits of exercise, GGT, HDL-C, TC, TG, FPG, HbA1c, smoking status, drinking status, SBP and height^
^Wang X., 2024^ [43]	^China^	^Cross-sectional^	^General population^	^1384 (525)^	^388^	^996^	^Ultrasound^	^NR^	^42.4 (11.7)^	^Age, gender, body mass index (only for TyG), SBP, hemoglobin, and ALT^
^Xue, 2022^ [44]	^China (US population)^	^Cross-sectional^	^General population (NHANES)^	^1727 (902)^	^NAFLD: 737^	^990^	^Transient elastography+MAFLD criteria^	^> 18 years^	^53.00 (36.00-65.00)^	^Age, gender, PIR, education level, smoking status, diabetes, hypertension, and obesity or overweight^
					^MAFLD: 718^	^1009^	^Transient elastography^			
^Yang, 2025^ [45]	^China^	^Cross-sectional^	^General population^	^71299 (33965)^	^24283^	^47016^	^Ultrasound+MAFLD criteria^	^NR^	^MAFLD: 49 (38–56); non-MAFLD: 41 (32–52)^	^Sex, age, BMI, HTN, diabetes mellitus, AST/ALT, AST, ALT, TC, GGT, HDL-C, and LDL-C^
					^M:17487^	^M:19847^				
^Yang, 2023^ [46]	^China^	^Cross-sectional^	^General population^	^7968 (553)^	^3268^	^4700^	^Ultrasound+MAFLD criteria^	^NR^	^39.60 (10.96)^	^NA^
^Zeng, 2023^ [47]	^China^	^Cross-sectional^	^General population^	^2148 (535)^	^354^	^1794^	^Ultrasound^	^NR^	^non-NAFLD:57 (53–63); NAFLD: 58 (53–61)^	^Age, sex, history of HTN^
^Zhang, 2017^ [48]	^China^	^Cross-sectional^	^Non-obese^	^6809 (2751)^	^1630^	^5179^	^Ultrasound^	^>20 years^	^48.4(15.1)^	^Age and sex, DBP, HDL, ALT, uric acid, WBC, BMI^
^Zou, 2023^ [49]	^China (US population)^	^Cross-sectional^	^General population (NHANES and western China)^	^NHANES:7398 (3746)^	^NHNAES: 3749^	^NHNAES: 3649^	^Transient elastography+MAFLD criteria^	^NR^	^NHNAES: 48.96 (18.06)^	^NA^
				^China: 4880 (1942)^	^M: 1999^	^M: 1653^			^China: 44.15(12.14)^	
					^China: 1552^	^China: 3328^				
					^M: 1249^	^M: 1689^				

ALB, Albumin; ALP, Alkaline phosphatase; ALT, Alanine aminotransferase; AST, Aspartate aminotransferase; BMI, Body Mass Index; BUA, Blood uric acid; CI, Confidence Interval; Cr, Creatinine; CT, Computed Tomography; DBIL, Direct bilirubin; DBP, Diastolic blood pressure; DL, Dyslipidemia; DM, Diabetes Mellitus; eGFR, Estimated Glomerular Filtration Rate; F, Female; FIPR, Family Income-to-Poverty Ratio; FPG, Fasting plasma glucose; GGT, Gamma-glutamyl transferase; GLB, Globulin; HbA1c, Hemoglobin A1c; HDL-C, High-density lipoprotein cholesterol; HOMA-IR, Homeostasis model assessment of insulin resistance; HTN, Hypertension; IQR, Interquartile range; LDL-C, Low-density lipoprotein cholesterol; MAFLD, Metabolic-associated fatty liver disease; M, Male; NA, Not available; NAFLD, Non-alcoholic fatty liver disease; NHANES, National Health and Nutrition Examination Survey; NR, Not reported; PIR, Poverty income ratio; RHR, Resting heart rate; ROC, Receiver operating characteristic; SBP, Systolic blood pressure, TG, Triglycerides; TC, Total Cholesterol; UA, Uric Acid; WBC, White Blood Cell

**Table 2:** Effect size of the included studies.

Author, year	Study outcome	Outcome assessment	Value	Other findings
Bockarie, 2023	NAFLD	TyG-BMI mean NAFLD	142.61 (44.47)	In the all-male group, TyG-BMI and FLI were the most accurate predictors of NAFLD.
		TyG-BMI mean non-NAFLD	110.16 (34.71)	
		aOR	1.00 (0.98-1.03)	
		sen	0.14	
		spe	0.9742	
		AUC	0.78 (0.71-0.85)	
Chang, 2023	MAFLD	aOR	1.073 (1.070-1.075)	TyG-BMI demonstrated the strongest predictive value in both the female and lean groups (BMI < 23 kg/m²). It outperformed TyG-WC, BMI, WC, and TyG in predicting MAFLD.
		sen (M)	0.799	
		sen (F)	0.907	
		spe (M)	0.763	
		spe (F)	0.812	
		AUC (M)	0.870 (0.864-0.876)	
		AUC (F)	0.933 (0.927-0.938)	
		aOR Q4 vs. Q1	380.87 (263.23-551.05)	
		TyG-BMI mean MAFLD	202.04 (28.85)	
		TyG-BMI mean non-MAFLD	154.16 (24.91)	
Chen, 2024	MAFLD	aOR	1.044 (1.022-1.065)	There is a significant association between elevated SUA/Cr levels and an increased risk of MAFLD in the Chinese adult population, with TyG-BMI mediating this relationship.
Gao, 2022	MAFLD	TyG-BMI median MAFLD	212.551 (202.356-225.156)	TyG/HDL-C was superior to TyG-BMI in predicting MAFLD.
		TyG-BMI median non-MAFLD	203.00 (180.248-211.570)	
		aOR	1.231 (1.051-1.442)	
		sen	0.912	
		spe	0.692	
		AUC	0.812 (0.744-0.880)	
Han, 2024	MAFLD	TyG-BMI mean MAFLD	257.51 (31.99)	The AUC values increased in the following order: TyG index < TyG-BMI < TyG-WC. Women exhibited higher AUC values than men across all categories.
		TyG-BMI mean non-MAFLD	195.92 (28.72)	
		AUC	0.938 (0.909-0.968)	
		AUC (F)	0.940 (0.849-1.000)	
		AUC (M)	0.928 (0.893-0.963)	
Hu, 2022	NAFLD	TyG-BMI mean NAFLD	218.162 (30.871)	TyG-BMI has a higher diagnostic accuracy in BMI < 24 compared to BMI > 24
		TyG-BMI mean non-NAFLD	168.514 (27.726)	
		AUC	0.888 (0.879-0.897)	
		sen	0.8475	
		spe	0.7798	
		AUC (M)	0.84 (0.83–0.86)	
		sen (M)	0.911	
		spe (M)	0.554	
		AUC (F)	0.92 (0.92–0.93)	
		sen (F)	0.822	
		spe (F)	0.846	
Hu, 2025	MASLD	TyG-BMI median non-MASLD	33.73 (22.08-45.38)	The AUC values tended to increase in the following order: HOMA-IR < VAI < TyG-BMI < LAP
		TyG-BMI median MASLD	53.37 (37.6-69.14)	
		aOR Q4 vs. Q1	17.009 (11.870-24.373)	
		sen	0.626	
		spe	0.763	
		AUC	0.765 (0.743-0.787)	
Khamseh, 2021	NAFLD	aOR	1.01 (1.000-1.029)	TyG-WC was a significant predictor of NAFLD, with the highest standardized OR. In contrast, liver fibrosis was more strongly associated with TyG-BMI.
		AUC	0.675 (0.598–0.752)	
		sen	0.783	
		spe	0.513	
		TyG-BMI mean non-NAFLD	256.0 (30.3)	
		TyG-BMI mean NAFLD	276.4 (30.3)	
Khamesh, 2024	MAFLD	TyG-BMI median MAFLD	282.15 (259.90–310.30)	TyG-WC, TyG-BMI, and TyG-WHtR were the most effective predictors of MAFLD. Insulin-based measures performed better in detecting advanced fibrosis.
		TyG-BMI median non-MAFLD	253.23 (227.13–277.98)	
		AUC	0.737 (0.699–0.775)	
		sen	0.7969	
		spe	0.5463	
Kilonzo, 2024	NAFLD	TyG-BMI median NAFLD	320.2 (289.1–354.6)	We found insufficient evidence to recommend using TyG and TyG-BMI as surrogates for hepatic ultrasound in detecting NAFLD.
		TyG-BMI median non-NAFLD	286.4 (266.1–312.3)	
		AUC	0.711 (0.64–0.78)	
		sen	0.6	
		spe	0.754	
		aOR (TyG-BMI ≥ 293.4)	1.9 (0.8–4.7)	
Kim, 2023	MAFLD	TyG-BMI mean MAFLD	237.5 (34.7)	TyG-BMI was superior to TyG-WC in predicting MAFLD.
		TyG-BMI mean non-MAFLD	189.3 (30.4)	
		aOR Q4 vs. Q1	128.592 (100.601-164.371)	
		AUC	0.867 (0.862-0.872)	
		sen	0.863	
		spe	0.723	
		AUC (M)	0.812 (0.803-0.820)	
		sen (M)	0.819	
		spe (M)	0.651	
		AUC (F)	0.895 (0.888-0.901)	
		sen (F)	0.889	
		spe (F)	0.764	
Kim, 2021	NAFLD	TyG-BMI mean non-NAFLD	205.2 (27.5)	The AUC values tended to increase in the following order: HOMA-IR < TyG < TyG-BMI < TyG-WC
		TyG-BMI mean NAFLD	243.7 (29.1)	
		aOR Q4 vs. Q1	25.34 (19.93-32.23)	
		AUC	0.837 (0.830–0.844)	
Lee, 2024	MASLD	AUC	0.898 (0.880-0.915)	The AUC values tended to increase in the following order: VAI < LAP < DSI < HIS = TyG-BMI < FSI < FLI < ZJU
		TyG-BMI mean non-MASLD	200.5 (30.6)	
		TyG-BMI mean MASLD	265.6 (41.8)	
Li D., 2024	NAFLD	aOR	1.038(1.035-1.040)	The AUC values tended to increase in the following order: WC < BMI < TyG < TyG-WC < TyG-BMI
		AUC	0.856 (0.848-0.865)	
		sen	0.869	
		spe	0.681	
		AUC (M)	0.807 (0.794–0.819)	
		sen (M)	0.738	
		spe (M)	0.736	
		AUC (F)	0.883 (0.868–0.897)	
		sen (F)	0.863	
		spe (F)	0.744	
		TyG-BMI mean non-NAFLD	161.18 (43.12)	
		TyG-BMI mean NAFLD	208.99 (44.02)	
Li H., 2023	NAFLD	aOR Q4 vs. Q1	16.7 (15.1-18.53)	The AUC values tended to increase in the following order: VAI < TG/HDL < TyG < LAP < HIS<METS-IR < TyG-BMI
		aOR Q5 vs. Q1	43.02 (38.89-47.72)	
		AUC	0.8059 (0.8025-0.8094)	
		sen	0.7562	
		spe	0.704796	
		TyG-BMI mean NAFLD	252.77 (33.83)	
		TyG-BMI mean non-NAFLD	212.92 (33.36)	
Li N., 2022	NAFLD	aOR Q4 vs. Q1	4.868 (2.576–9.200)	Compared to the TyG index, TG/HDL-C, and HOMA-IR, TyG-BMI was a more effective predictor of NAFLD in T2D.
		AUC	0.727 (0.691–0.764)	
		sen	0.622	
		spe	0.738	
		AUC (M)	0.739 (0.695–0.783)	
		sen (M)	0.743	
		spe (M)	0.631	
		AUC (F)	0.702 (0.636–0.768)	
		sen (F)	0.737	
		spe (F)	0.617	
Li S., 2023	NAFLD	TyG-BMI mean non-NAFLD	199.8 (196.7-203.0)	In the non-obese population, the indices of TyG, TyG-BMI, TyG-WC, and TG/HDL-C were positively correlated with CAP and NAFLD. Among these indices, TyG-WC was superior in identifying NAFLD.
		TyG-BMI mean NAFLD	234.2 (230.2-238.2)	
		AUC	0.789 (0.768-0.810)	
		sen	0.751	
		spe	0.698	
		aOR	1.032 (1.019-1.045)	
Li Y., 2020	NAFLD	AUC	0.8489 (0.8375-0.8603)	Gender and TyG-BMI had a significant interaction with NAFLD incidence.
		sen	0.8205	
		spe	0.7348	
		HR TyG-BMI (per SD)	3.089 (2.628-3.631)	
		HR Q4 vs. Q1	38.242 (12.065-121.213)	
Liu, 2022	MAFLD	aOR	1.215 (1.142-1.293)	In identifying MAFLD, TyG, TyG-BMI, TG, and TG/HDL-C were found to be the most vital indices based on the random forest method, with an AUC greater than 0.9.
		AUC	0.956 (0.933-0.980)	
		sen	0.918	
		spe	0.886	
		AUC (M)	0.945 (0.912-0.978)	
		sen (M)	0.907	
		spe (M)	0.875	
		AUC (F)	0.978 (0.951-1.000)	
		sen (F)	1	
		spe (F)	0.904	
		TyG-BMI mean non-MAFLD	189.87 (28.31)	
		TyG-BMI mean MAFLD	260.07 (38.91)	
Malek, 2021	NAFLD	TyG-BMI median non-NAFLD	249.81 (220.75–281.61)	TyG-related indices, which incorporate TyG and obesity parameters, are more effective at identifying hepatic steatosis than TyG alone
		TyG-BMI median NAFLD	284.00 (262.50–318.35)	
		aOR Q4 vs. Q1	6.75 (1.49–30.67)	
		AUC	0.751 (0.670–0.833)	
Otsubo, 2023	NAFLD	AUC	0.844 (0.838-0.850)	In both men and women, the FLI and TyG-BMI demonstrated significantly higher AUC values for predicting NAFLD compared to other indicators.
		sen	0.769	
		spe	0.764	
		AUC (M)	0.783 (0.775-0.792)	
		sen (M)	0.708	
		spe (M)	0.722	
		AUC (F)	0.868 (0.856-0.880)	
		sen (F)	0.816	
		spe (F)	0.762	
		aOR (per SD)	2.8 (2.6-3.0)	
Peng, 2023	MAFLD	aOR (quartile increment)	17.118 (7.165-40.895)	For females, TyG-WHtR showed the best performance in identifying MAFLD and NAFLD, with AUCs of 0.845 and 0.831, respectively. For males, TyG-WC demonstrated the best performance in identifying MAFLD and NAFLD, with AUCs of 0.900 and 0.855, respectively.
		TyG-BMI median non-MAFLD	200.81 (174.19-227.81)	
		TyG-BMI median MAFLD	270.06 (240.02-314.40)	
		AUC	0.867 (0.841-0.889)	
		sen	0.7221	
		spe	0.8828	
		AUC (M)	0.896 (0.863-0.924)	
		sen (M)	0.7962	
		spe (M)	0.8645	
		AUC (F)	0.843 (0.804-0.878)	
		sen (F)	0.7069	
		spe (F)	0.8634	
	NAFLD	TyG-BMI median non-NAFLD	202.30 (174.18-231.15)	
		TyG-BMI median NAFLD	266.78 (236.94-312.21)	
		aOR (quartile increment)	8.113 (4.084-16.116)	
		AUC	0.836 (0.808-0.861)	
		sen	0.7323	
		spe	0.8196	
		AUC (M)	0.849 (0.811-0.883)	
		sen (M)	0.773	
		spe (M)	0.8127	
		AUC (F)	0.830 (0.789-0.865)	
		sen (F)	0.6982	
		spe (F)	0.8448	
Priego-Parra, 2024	MASLD	TyG-BMI median non-MASLD	229.2 (211–262.8)	After accounting for sociodemographic variables, the TyG-WC index was identified as the most effective predictor of MASLD.
		TyG-BMI median MASLD	294.5 (262–324.7)	
		AUC	0.82 (0.73–0.89)	
		sen	0.745	
		spe	0.743	
		AUC (M)	0.80 (p value 0.003)	
		sen (M)	0.754	
		spe (M)	0.777	
		AUC (F)	0.82 (p value 0.003)	
		sen (F)	0.739	
		spe (F)	0.766	
Sheng, 2021	NAFLD	aOR (per SD) M	4.31 (3.88-4.79)	Their data indicate that TyG index-related parameters, along with LAP, HSI, BMI, and WC, are good predictors of NAFLD. Among these, TyG index-related parameters demonstrated the highest predictive potential.
		aOR (per SD) F	4.62 (3.97-5.38	
		AUC	0.8862 (0.8797-0.8927)	
		sen	0.8381	
		spe	0.7787	
		AUC (M)	0.8428 (0.8331-0.8525)	
		sen (M)	0.8703	
		spe (M)	0.8071	
		AUC (F)	0.9084 (0.8964-0.9204)	
		sen (F)	0.7723	
		spe (F)	0.7551	
Song, 2022	NAFLD	aOR Q4 vs. Q1	26.815 (22.884–31.422)	The ORs for NAFLD increased across all indices, with TyG-WC (OR: 39.251) and FLI (OR: 38.937) showing the highest values.
		AUC	0.827 (0.821–0.834)	
		AUC (M)	0.774 (0.763–0.786)	
		AUC (F)	0.832 (0.822–0.841)	
Tian, 2024	NAFLD	TyG-BMI median NAFLD	56.98 (43.92-74.61)	TyG-BMI shows promise as a predictor of NAFLD + T2DM, especially among lean male patients.
		TyG-BMI median non-NAFLD	38.41 (26.43-56.39)	
		AUC	0.738 (0.617–0.778)	
		AUC (M)	0.764 (0.691—0.827)	
		sen (M)	0.9032	
		spe (M)	0.4789	
		AUC (F)	0.702 (0.605—0.788)	
		sen (F)	0.9038	
		spe (F)	0.4423	
		aOR	1.038 (1.014–1.062)	
Wang M., 2023	NAFLD	aOR	1.039 (1.031–1.046)	The TyG-BMI demonstrated greater accuracy in predicting NAFLD compared to the TyG and showed a stronger association with the severity of hepatic steatosis.
		AUC	0.808	
		sen	0.8331	
		spe	0.6313	
		AUC (M)	0.778	
		AUC (F)	0.863	
Wang R., 2021	NAFLD	aOR (per SD)	3.90 (3.54-4.29)	The TyG-BMI proved to be a superior predictor of NAFLD risk compared to other traditional indicators.
		aOR Q4 vs. Q1	25.52 (13.36-48.74)	
		aOR Q5 vs. Q1	74.76 (38.86-143.79)	
		AUC	0.886 (0.8797–0.8927)	
		sen	0.8381	
		spe	0.7787	
Wang X., 2024	NAFLD	TyG-BMI mean non-NAFLD	195.61 (30.42)	Both TyG and TyG-BMI were associated with an increased risk of NAFLD among individuals living in high-altitude regions, with TyG-BMI demonstrating superior predictive capability.
		TyG-BMI mean NAFLD	246.13 (30.32)	
		aOR Q4 vs. Q1	48.55 (25.12–93.81)	
		AUC	0.883 (0.864–0.902)	
		sen	0.832	
		spe	0.78	
Xue, 2022	NAFLD	AUC	0.804 (0.784–0.822)	TyG-WC, TyG-WHtR, and TyG-BMI are valuable tools for the early screening of NAFLD and MAFLD. These parameters, along with HOMA-IR, are better suited for evaluating metabolic risks and monitoring disease progression in patients with NAFLD.
		sen	0.7938	
		spe	0.6909	
		aOR Q4 vs. Q1	26.661 (17.685-40.193)	
		TyG-BMI median NAFLD	276.50 (242.85–323.78)	
		TyG-BMI median non-NAFLD	216.20 (184.29–249.33)	
	MAFLD	AUC	0.822 (0.803–0.840)	
		sen	0.8148	
		spe	0.6967	
		TyG-BMI median non-MAFLD	176.32 (161.74-193.57)	
		TyG-BMI median MAFLD	255.49 (226.35-298.87)	
Yang, 2025	MAFLD	TyG-BMI median non-MAFLD	121.79 (107.57-137.40)	TyG index and related parameters had greater predictive value in the female, younger, and BMI < 23.7 populations.
		TyG-BMI median MAFLD	164.82 (149.79-182.71)	
		aOR	1.059 (1.056-1.061)	
		AUC	0.92 (0.91-0.92)	
		sen	0.8	
		spe	0.87	
		AUC (M)	0.89 (0.88-0.89)	
		sen (M)	0.77	
		spe (M)	0.83	
		AUC (F)	0.93 (0.93-0.93)	
		sen (F)	0.82	
		spe (F)	0.89	
Yang, 2023	MAFLD	TyG-BMI mean non-MAFLD	195.58 (30.00)	TyG-BMI, BMI, TyG, TG/HDL-C, and TG ranked as the top five important indicators, with TyG-BMI demonstrating the highest predictive accuracy for MAFLD based on the ROC curve.
		TyG-BMI mean MAFLD	248.17 (34.38)	
		AUC	0.884 (0.877-0.891)	
		sen	0.817	
		spe	0.783	
Zeng, 2023	NAFLD	TyG-BMI median non-NAFLD	207.24 (187.08-226.14)	TyG-BMI and HOMA-IR can be used to detect non-obese and obese NAFLD, respectively, showing superior detection ability compared to other IR markers.
		TyG-BMI median NAFLD	236.65 (221.16-257.10)	
		AUC	0.788 (0.766–0.811)	
		sen	0.842	
		spe	0.623	
		aOR Q4 vs. Q1	45.527 (20.842-99.447)	
Zhang, 2017	NAFLD	TyG-BMI median NAFLD	205.2 (193.9–217.7)	Comparison of AUROC using Delong test: TyG-BMI > BMI > TyG > TG > FPG
		TyG-BMI median non-NAFLD	176.7 (160.6–192.7)	
		aOR Q4 vs. Q1	15.3 (9.8–23.9)	
		aOR (per SD)	3.4 (3.0–3.9)	
		AUC	0.835 (0.824–0.845)	
		AUC (M)	0.814 (0.800–0.828)	
		AUC (F)	0.855 (0.838–0.872)	
Zou, 2023	MAFLD (NHANES)	TyG-BMI mean non-MAFLD	218.59 (50.10)	TyG-WC was superior in the NAHANES cohort and TyG-BMI in the Western China cohort.
		TyG-BMI mean MAFLD	296.81 (65.24)	
		AUC	0.850 (0.841–0.859)	
		sen	0.819	
		spe	0.727	
		AUC (M)	0.865 (0.853-0.876)	
		sen (M)	0.809	
		spe (M)	0.765	
		AUC (F)	0.842 (0.830-0.855)	
		sen (F)	0.825	
		spe (F)	0.699	
	MAFLD (China cohort)	TyG-BMI mean non-MAFLD	186.00 (29.41)	
		TyG-BMI mean MAFLD	239.05 (29.79)	
		AUC	0.903 (0.895–0.911)	
		sen	0.836	
		spe	0.811	
		AUC (M)	0.859 (0.847-0.872)	
		sen (M)	0.849	
		spe (M)	0.716	
		AUC (F)	0.944 (0.933-0.954)	
		sen (F)	0.931	
		spe (F)	0.829	

AUC, Area Under the Curve; aOR, adjusted Odds Ratio; BMI, Body Mass Index; CAP, Controlled Attenuation Parameter; Cr, Creatinine; FLI, Fatty Liver Index; HOMA-IR, Homeostasis Model Assessment of Insulin Resistance; HR, Hazard Ratio; LAP, Lipid Accumulation Product; MAFLD, Metabolic Dysfunction-associated Fatty Liver Disease; MASLD, Metabolic Dysfunction-associated Steatotic Liver Disease; NAFLD, Non-Alcoholic Fatty Liver Disease; Q, Quartile; sen, Sensitivity; spe, Specificity; TyG, Triglyceride-glucose index; TyG-BMI, Triglyceride-glucose index-Body Mass Index; TyG-WC, Triglyceride-glucose index-Circumference; TyG-WHtR, Triglyceride-glucose index-Waist-to-Height Ratio; WC, Waist Circumference; ZJU, ZJU index.

### The mean difference of TyG-BMI in MASLD vs. non-MASLD groups

**Fig 2:**
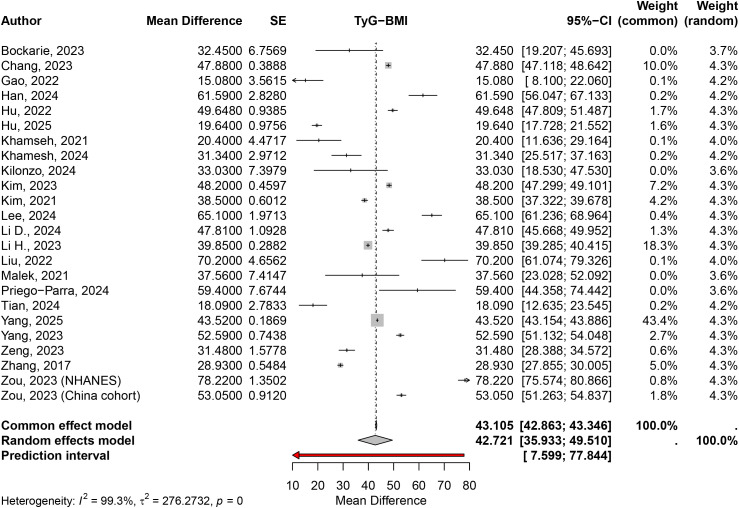
The mean difference of TyG-BMI in MASLD vs. non-MASLD groups.

The random effects model for the mean difference analysis included 24 studies and yielded a pooled mean difference of 42.7214 (95% CI: 35.9331–49.5096; z = 12.33; p < 0.0001). Substantial heterogeneity was observed (I² = 99.3%, Q = 3185.45, df = 23, p < 0.0001). Patients with MASLD have a significantly higher TyG-BMI compared to patients without MASLD (Fig 2).

In the subgroup analysis by disease definition, 11 studies focused on NAFLD with a mean difference of 34.6765 (95% CI: 28.4465–40.9065; τ² = 97.6531; τ = 9.8820), while 13 studies on MAFLD reported a mean difference of 49.5614 (95% CI: 39.3799–59.7428; τ² = 342.3150; τ = 18.5018). The test for subgroup differences revealed significant heterogeneity between the two groups (Q = 5.97, df = 1, p = 0.0145). TyG-BMI mean difference was significantly higher in the MAFLD group compared to the NAFLD group (S1 Fig).

In the subgroup analysis by population type, 17 studies focusing on the general population reported a mean difference of 49.9946 (95% CI: 43.6121–56.3770; τ² = 172.4196; τ = 13.1309). For the T2DM subgroup (4 studies), the mean difference was 19.4570 (95% CI: 17.7224–21.1917; τ² < 0.0001; τ = 0.0015). The subgroup of overweight participants and patients with obesity (2 studies) yielded a mean difference of 25.3400 (95% CI: 13.2598–37.4202; τ² = 42.3958; τ = 6.5112), while the non-obese subgroup (2 studies) had a mean difference of 31.0773 (95% CI: 25.8420–36.3126; τ² = 11.4452; τ = 3.3831). The test for subgroup differences indicated significant heterogeneity between subgroups (Q = 93.63, df = 3, p < 0.0001) (S2 Fig).

### The odds ratio for the association of TyG-BMI with MASLD

**Fig 3:**
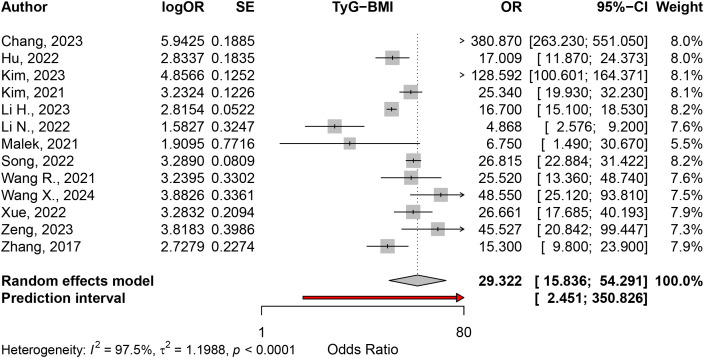
The odds ratio of TyG-BMI fourth quartile vs. first quartile.

**Fig 4:**
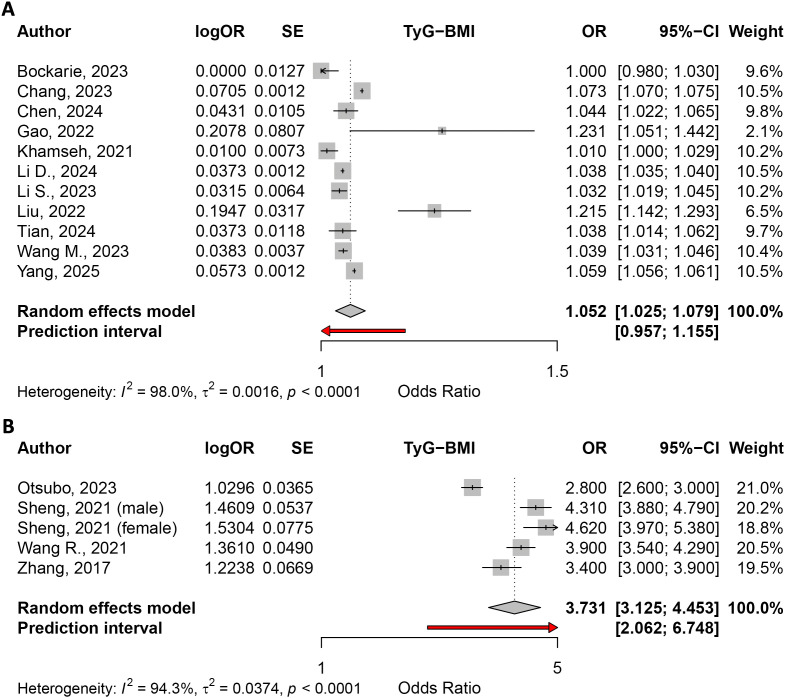
(A) The odds ratio for a 1-unit increment in TyG-BMI; (B) the odds ratio for a 1-SD increment in TyG-BMI.

The random effects model for the odds ratio (OR) of TyG-BMI fourth quartile vs. first quartile meta-analysis, based on 13 studies, yielded an odds ratio of 29.3217 (95% CI: 15.8361–54.2914; z = 10.75; p < 0.0001). The test of heterogeneity was significant (I² = 97.5%, Q = 483.48, df = 12, p < 0.0001) (Fig 3). For a 1-unit increment, the random effects model included 11 studies and reported an OR of 1.0517 (95% CI: 1.0252–1.0788; z = 3.87; p = 0.0001, I² = 98.0%, Q = 495.27, df = 10, p < 0.0001). (Fig 4A). For a 1-SD increment, based on 5 studies, the random effects model reported an OR of 3.7306 (95% CI: 3.1255–4.4529; z = 14.58; p < 0.0001). The test of heterogeneity was also significant (I² = 94.3%, Q = 70.19, df = 4, p < 0.0001). (Fig 4B).

### Sensitivity and specificity of TyG-BMI for diagnosing MASLD

**Fig 5:**
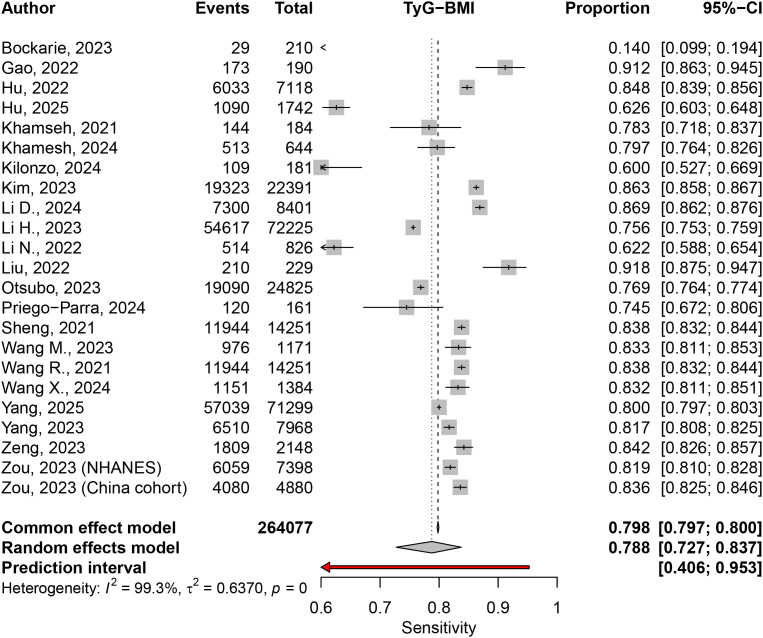
Sensitivity of TyG-BMI for diagnosing MASLD.

The sensitivity meta-analysis, based on 23 studies, revealed a pooled sensitivity of 0.7876 (95% CI: 0.7274–0.8375) under the random effects model (I² = 99.3%) (Fig 5). Subgroup analysis by disease definition indicated a sensitivity of 0.7548 (95% CI: 0.6493–0.8365) for NAFLD and 0.8229 (95% CI: 0.7722–0.8643) for MAFLD. However, the test for subgroup differences was not significant (Q = 1.82, p = 0.1776) (S3 Fig).

**Fig 6:**
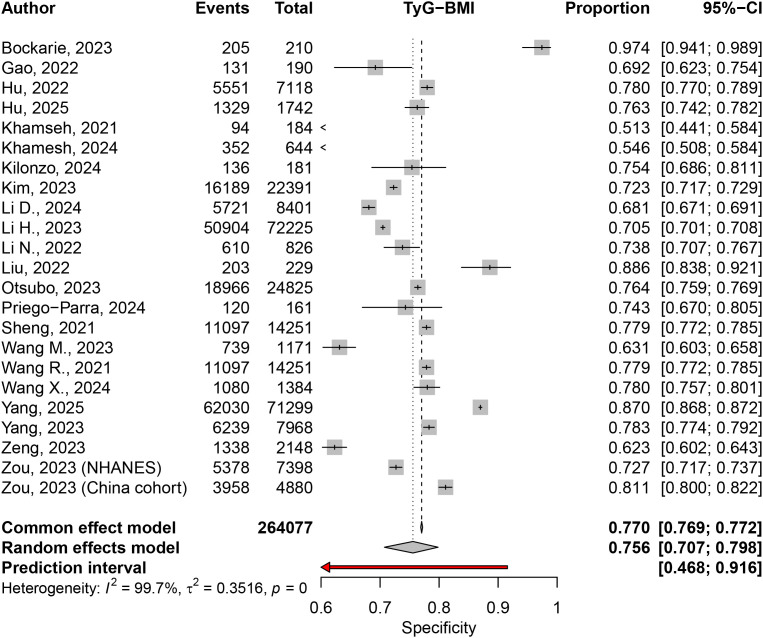
Specificity of TyG-BMI for diagnosing MASLD.

The specificity meta-analysis, based on the same 23 studies reported a pooled specificity of 0.7557 (95% CI: 0.7074–0.7983) under the random effects model (I² = 99.7%) (Fig 6). Subgroup analysis by disease definition showed specificities of 0.7492 (95% CI: 0.6726–0.8128) for NAFLD and 0.7654 (95% CI: 0.7050–0.8167) for MAFLD, with no significant subgroup differences (Q = 0.13, p = 0.7224) (S4 Fig).

Subgroup analysis by sex indicated a sensitivity of 0.8117 (95% CI: 0.7772–0.8419) for males and 0.8677 (95% CI: 0.8222–0.9029) for females under the random effects model, with significant subgroup differences (Q = 4.05, p = 0.0441). Specificity results were 0.7487 (95% CI: 0.6941–0.7963) for males and 0.7498 (95% CI: 0.6801–0.8086) for females, with no significant subgroup differences (Q = 0.00, p = 0.9787) (S5 and S6 Figs).

Subgroup analysis by population type showed a sensitivity of 0.8034 (95% CI: 0.7340–0.8583) for the general population, 0.7488 (95% CI: 0.5325–0.8864) for individuals with T2DM, 0.6984 (95% CI: 0.5568–0.8102) for overweight participants and patients with obesity, and 0.7824 (95% CI: 0.7464–0.8145) for non-obese population under the random effects model, with no significant differences between subgroups (Q = 2.51, p = 0.4733). Specificity results were 0.7709 (95% CI: 0.7111–0.8215) for the general population, 0.7463 (95% CI: 0.7173–0.7733) for T2DM, 0.6416 (95% CI: 0.4596–0.7903) for overweight participants and patients with obesity, and 0.7346 (95% CI: 0.7038–0.7633) for non-obese populations, with no significant subgroup differences (Q = 2.86, p = 0.4142) (S7 and S8 Figs).

### Area under the curve for TyG-BMI in MASLD prediction

**Fig 7:**
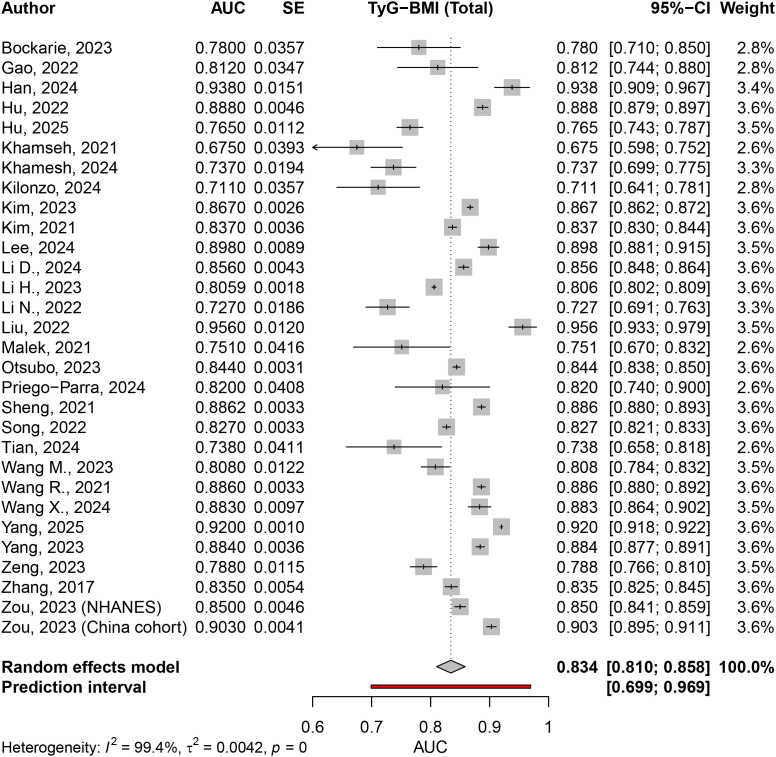
The area under the curve for TyG-BMI in MASLD prediction.

The pooled analysis of 30 studies assessing the association between the TyG-BMI and MASLD demonstrated high diagnostic performance, with an overall AUC of 0.8343 (95% CI: 0.8101, 0.8584; p < 0.0001). However, significant heterogeneity was observed across the studies, with an I² value of 99.4%, τ² of 0.0042, and a heterogeneity test p-value < 0.0001 (Fig 7).

In the sex-specific subgroup analysis, the pooled AUC for the male population, based on 17 studies, was 0.8323 (95% CI: 0.8053, 0.8592; p < 0.0001), with substantial heterogeneity (I² = 98.8%, τ² = 0.0029). Similarly, for the female population, also based on 17 studies, the pooled AUC was higher at 0.8770 (95% CI: 0.8445, 0.9095; p < 0.0001), with significant heterogeneity (I² = 98.0%, τ² = 0.0043). A comparison of AUCs between males and females revealed a statistically significant difference (Q = 4.31, p = 0.0378). TyG-BMI had a better diagnostic performance in females compared to males for MASLD (S9 Fig).

The analysis also evaluated differences based on disease definition. For studies investigating MAFLD, the pooled AUC from 12 studies was 0.8650 (95% CI: 0.8266, 0.9034; p < 0.0001), with high heterogeneity (I² = 98.7%, τ² = 0.0043). In contrast, for studies focusing on NAFLD, the pooled AUC from 18 studies was slightly lower at 0.8147 (95% CI: 0.7868, 0.8425; p < 0.0001), with similarly high heterogeneity (I² = 98.3%, τ² = 0.0033). The test for subgroup differences between MAFLD and NAFLD showed statistically significant results (Q = 4.32, p = 0.0377). TyG-BMI had a better diagnostic performance for patients with MAFLD compared to NAFLD (S10 Fig).

Subgroup analysis by population type further revealed variations in the diagnostic performance of the TyG-BMI index. For the general population, comprising 21 studies, the pooled AUC was 0.8604 (95% CI: 0.8376, 0.8831; p < 0.0001), with high heterogeneity (I² = 99.5%, τ² = 0.0027). Among patients with T2DM, the pooled AUC from five studies was lower at 0.7564 (95% CI: 0.7302, 0.7825; p < 0.0001), with minimal heterogeneity (I² = 31.6%, τ² = 0.0003). For patients with obesity or overweight populations, based on two studies, the pooled AUC was 0.6947 (95% CI: 0.6429, 0.7465; p < 0.0001), with no heterogeneity (I² = 0%). In the non-obese population, based on four studies, the pooled AUC was 0.8305 (95% CI: 0.8053, 0.8557; p < 0.0001, I² = 89.1%,). The test for subgroup differences by population type was statistically significant (Q = 56.49, p < 0.0001) (S11 Fig).

### Risk of bias assessment

Independent investigators evaluated the quality of the studies included in the review using the modified Newcastle-Ottawa Scale (NOS), specific to cohort, case-control, and cross-sectional studies. The potential for bias in these studies was minimal. Quality assessments are presented in Table 3. Most studies received high scores, indicating good quality, while only one scored as fair. None were classified as having a “poor” score.

**Table 3:** Risk of bias assessment.

Author, year	Selection					Comparability	Outcome		Overall score
	*Representativeness of the sample*	*Sample size*	*Non response rate*	*Ascertainment of the exposure/surveillance tool*			*Assessment of outcome*	*clearly described and appropriate statistical test*	
**Bockarie AS, 2023**	+	+	+	++		++	++	+	**10**
**Chang M, 2023**		+		++		++	++	+	**8**
**Gao Q, 2023**				++		++	++	+	**7**
**Han AL, 2024**	+	+		++			++	+	**7**
**Hu H, 2022**	+	+		++			++	+	**7**
**Hu M, 2025**		+		++		++	++	+	**8**
**Khamseh ME, 2021**	+		+	++		++	++	+	**9**
**Khamseh ME, 2024**	+	+		++		++	++	+	**9**
**Kilonzo SB, 2024**	+			++		++	++	+	**8**
**Kim AH, 2023**	+	+		++		++	++	+	**9**
**Kim HS, 2021**	+	+		++		++	++	+	**9**
**Lee J, 2024**	+	+		++			++	+	**7**
**Li, D, 2024**	+	+		++		++	++	+	**9**
**Li H, 2023**		+	+	++		++	++	+	**9**
**Li N, 2022**		+		++		++	++	+	**8**
**Li S, 2023**	+	+	+	++		++	++	+	**10**
**Liu Z, 2022**		+	+	++		++	++	+	**9**
**Malek M, 2021**			+	++		++	++	+	**8**
**Otsubo N, 2022**	+	+		++		++	++	+	**9**
**Peng H, 2023**	+	+		++		++	++	+	**9**
**Priego-Parra BA, 2024**	+		+	++		++	++	+	**9**
**Sheng G, 2021**	+	+		++		++	++	+	**9**
**Song S, 2022**		+	+	++		++	++	+	**9**
**Tian J, 2024**		+	+	++		++	++	+	**9**
**Wang M, 2023**	+	+	+	++		++	++	+	**10**
**Wang R, 2021**	+	+		++		++	++	+	**9**
**Wang X, 2024**	+	+	+	++		++	++	+	**10**
**Xue Y, 2022**	+	+		++		++	++	+	**9**
**Yang X, 2025**	+	+		++		++	++	+	**9**
**Yang Z, 2023**		+	+	++		++	++	+	**9**
**Zeng P, 2023**	+	+		++		++	++	+	**9**
**Zhang S, 2017**		+		++		++	++	+	**8**
**Zou H, 2023**	+	+		++			++	+	**7**
**Author, year**	**Selection**				Comparability	**Outcome**			**Overall score**
	*Representativeness of exposure*	*Selection of the non- exposed*	*Ascertainment of exposure*	*Outcome of interest not present at start*		*Assessment of outcome*	*Adequate follow up length*	*Adequacy of follow up*	
**Li Y, 2020**	+	+	+		++	+	+	+	8
**Author, year**	**Selection**				Comparability	**Outcome**			**Overall score**
	*Adequate case definition*	*Representativeness of the cases*	*Selection of Controls*	*Definition of Controls*		*Ascertainment of exposure*	*same method for cases and controls*	*Non-Response rate*	
**Chen B, 2024**	+	+			++	+	+		**6**

**Fig 8:**
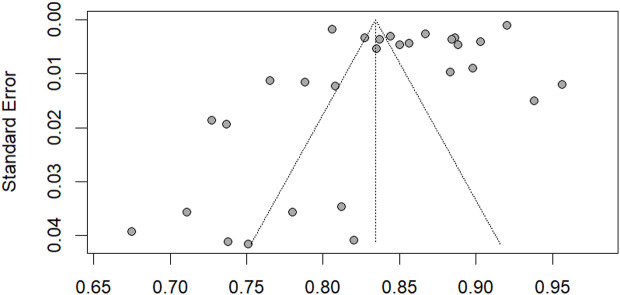
Funnel plot.

The funnel plot for publication bias is available in Fig 8. Egger’s regression test was conducted to evaluate funnel plot asymmetry. The test result indicated a t-value of −2.11 and a p-value of 0.0440, suggesting statistically significant evidence of publication bias (p < 0.05). The bias estimate was −6.3135, with a standard error (SE) of 2.9934. The multiplicative residual heterogeneity variance (tau^2) was 139.4794. Additionally, we used the rank correlation test by Begg and Mazumdar (1993) to further examine funnel plot asymmetry. The test result yielded a z-value of 0.38 with a p-value of 0.7076, indicating no statistically significant evidence of publication bias (p > 0.05). The bias estimate was 21.0000, with a standard error (SE) of 55.9911. The discrepancy between the two tests highlights the importance of using multiple methods to assess publication bias and interpreting the results with caution.

## Discussion

This systematic review and meta-analysis demonstrated a strong association between the TyG-BMI index and the fatty liver disease defined by NAFLD, MAFLD and MASLD. Individuals with these conditions had significantly higher TyG-BMI values compared to those without. The association was more pronounced in MAFLD than in NAFLD. Additionally, an increase in the TyG-BMI index was linked to higher odds of having MASLD. The diagnostic performance of TyG-BMI was notable, showing a relatively high sensitivity and specificity. Overall, the index demonstrated strong diagnostic accuracy, with better performance in females compared to males, in MAFLD compared to NAFLD, with the highest AUC value in the general population and the lowest value in patients with T2DM.

The TyG index was indeed first introduced by Simental-Mendía et al. in 2008 [50]. In their study, they explored the use of fasting triglyceride and glucose levels as a marker for insulin resistance, which is a key factor in metabolic disorders. The TyG index was shown to be a valuable surrogate for assessing insulin resistance in clinical practice, providing a more accessible alternative to more complex and expensive methods [50]. TyG-BMI is an emerging composite indicator that combines the TyG index, a reliable surrogate marker of insulin resistance, with BMI, a widely used measure of obesity. By integrating metabolic and anthropometric parameters, TyG-BMI provides a more comprehensive assessment of metabolic dysfunction compared to either marker alone [51].

Based on our findings, in almost all the included studies investigating the association of TyG-related indices, TyG-BMI was superior to TyG index in predicting MASLD; however, results regarding the superiority of TyG-BMI over TyG-WC and TyG-WHtR were inconsistent. The measured AUC in our study for the diagnostic performance of TyG-BMI in MASLD was higher than the diagnostic performance of the TyG index (0.834 vs. 0.75) in a study conducted by Wang et. al [52]. The TyG-BMI includes BMI, which reflects overall adiposity and body weight status, but the TyG index alone does not account for body composition or fat distribution, which are crucial in metabolic dysfunction. This makes TyG-BMI a more comprehensive indicator of metabolic health, as obesity or excess weight plays a critical role in metabolic disorders like MASLD [25]. Moreover, TyG-BMI has been shown in studies to correlate better with insulin resistance compared to the TyG index. Insulin resistance is a major contributor to conditions like MASLD, making TyG-BMI a stronger marker for identifying at-risk populations [53,54]. While our analysis focused on TyG-BMI, future research should directly compare the predictive performance of TyG-BMI against central obesity-adjusted indices (e.g., TyG-WC, TyG-WHtR) in well-characterized cohorts with standardized adiposity measurements. Pooled analyses of such studies may help resolve current controversies regarding optimal index selection.

Our subgroup analyses showed that TyG-BMI performed as a better predictor in females compared to males. Overall, females have a lower risk of MASLD compared to males [55]. However, in our systematic review, the mean/median age in most of the studies included was above 45 years. At this age, most women are undergoing menopause, characterized by declining estrogen levels. Research has shown that postmenopausal women have a prevalence of MASLD comparable to that of men, likely due to increased weight gain, fat redistribution, and dyslipidemia, all of which contribute to a higher risk of MASLD [55]. Furthermore, our findings underscore the differences in MASLD when the same increase in TyG-BMI is applied across different sexes. This may be attributed to sex-based variations in carbohydrate and lipid metabolism, as well as menopausal changes in body fat distribution and heightened susceptibility to metabolic complications [56].

The subgroup analyses by disease definition demonstrated that TyG-BMI performed as a better predictor in the MAFLD group compared to the NAFLD group. The diagnosis of MAFLD incorporates metabolic dysfunction as a primary criterion (e.g., insulin resistance, obesity, and metabolic syndrome), in addition to hepatic steatosis. TyG-BMI, which reflects both insulin resistance and obesity, aligns closely with these metabolic parameters [57]. Nevertheless, NAFLD criteria do not require the presence of metabolic dysfunction [58]. Thus, TyG-BMI may be less predictive in NAFLD because it primarily focuses on metabolic markers. Also, obesity is a core diagnostic criterion for MAFLD and is a strong driver of hepatic fat accumulation and systemic metabolic dysfunction [57]. Since TyG-BMI incorporates BMI, it directly captures the role of obesity in MAFLD.

The findings of this meta-analysis support the use of TyG-BMI as a non-invasive, cost-effective, and accessible marker for identifying individuals at risk of MASLD. Its strong association with insulin resistance and superior diagnostic performance, particularly in specific subgroups such as females and patients with MAFLD, highlights its potential for use in clinical settings. However, clinicians should consider population-specific factors, including age, sex, and metabolic profiles, when interpreting TyG-BMI values.

Our study was the first to determine the relationship between the TyG-BMI and MASLD. Moreover, the high number of included studies and subgroup analyses can provide good evidence and clues for further research. While this meta-analysis provides robust evidence for the association and diagnostic utility of TyG-BMI in MASLD, several limitations should be acknowledged. First, the included studies were heterogeneous in terms of population characteristics, study design, and diagnostic criteria for MASLD. Although the subgroup analyses partially addressed this issue, residual heterogeneity may affect the generalizability of the findings. Second, most studies were cross-sectional, limiting the ability to infer causality or assess the predictive value of TyG-BMI over time. Third, most studies were conducted on the Chinese population and few studies included diverse populations, such as those from low- and middle-income countries, where the burden of MASLD is rapidly increasing. Fourth, the cutoff values for the TyG-BMI varied among the included studies, which could result in differences in classifying individuals into high or low TyG-BMI groups. This variability may impact the observed associations with MASLD outcomes (for Q4 vs. Q1 OR and AUC). Fifth, BMI differences in different study populations were not investigated due to limitation in data. Lastly, differences in the adjustment for confounding factors, as well as inadequate adjustment, can result in biased estimates of the association. These limitations highlight the need for future research on the association of TyG-BMI with MASLD.

## Supporting information

S1 Table Search strategy. (DOCX)

S2 Table List of all studies identified in literature search. (DOCX)

S3 Table Excluded studies after full text review. (DOCX)

S1 File PRISMA checklist. (DOCX)

S1 Fig Mean difference of TyG-BMI in MASLD vs. non-MASLD patients in subgroups categorized by disease definition. (TIF)

S2 Fig Mean difference of TyG-BMI in MASLD vs. non-MASLD patients in subgroups categorized by population type. (TIF)

S3 Fig Sensitivity of TyG-BMI for diagnosing MASLD in subgroups categorized by disease definition. (TIF)

S4 Fig Specificity of TyG-BMI for diagnosing MASLD in subgroups categorized by disease definition. (TIF)

S5 Fig Sensitivity of TyG-BMI for diagnosing MASLD in subgroups categorized by sex. (TIF)

S6 Fig Specificity of TyG-BMI for diagnosing MASLD in subgroups categorized by sex. (TIF)

S7 Fig Sensitivity of TyG-BMI for diagnosing MASLD in subgroups categorized by population type. (TIF)

S8 Fig Specificity of TyG-BMI for diagnosing MASLD in subgroups categorized by population type. (TIF)

S9 Fig Area under the curve for TyG-BMI in MASLD prediction in subgroups categorized by sex. (TIF)

S10 Fig Area under the curve for TyG-BMI in MASLD prediction in subgroups categorized by disease definition. (TIF)

S11 Fig Area under the curve for TyG-BMI in MASLD prediction in subgroups categorized by population type. (TIF)
