# Supplementary material for: Association of triglyceride glucose-body mass index (TyG-BMI) with metabolic dysfunction-associated steatotic liver disease: A systematic review and meta-analysis
Source: PLoS One. 2025 Aug 4;20(8):e0324483. doi: 10.1371/journal.pone.0324483 (PMC12321072; doi:10.1371/journal.pone.0324483)
Supplement: S1 Table — (DOCX) [file pone.0324483.s001.docx]

**Table S1.** Search strategy

| **Database** | **Search query** | **Results (20 Jan 2025)** |
| --- | --- | --- |
| **PubMed** | (“triglyceride glucose-BMI”[tiab] OR “TyG-BMI”[tiab] OR “triglyceride glucose-body mass index”[tiab] OR “Triglyceride glucose body mass index”[tiab] OR “TyG-body mass index”[tiab] OR “TyG-BMI index”[tiab])  AND  (“Non-alcoholic Fatty Liver Disease”[MeSH] OR “Non-alcoholic Fatty Liver Disease”[tiab] OR “Non alcoholic Fatty Liver Disease”[tiab] OR “NAFLD”[tiab] OR “Nonalcoholic Fatty Liver Disease”[tiab] OR “Fatty Liver, Nonalcoholic”[tiab] OR “Fatty Livers, Nonalcoholic”[tiab] OR “Liver, Nonalcoholic Fatty”[tiab] OR “Livers, Nonalcoholic Fatty”[tiab] OR “Nonalcoholic Fatty Liver”[tiab] OR “Nonalcoholic Fatty Livers”[tiab] OR “Nonalcoholic Steatohepatitis”[tiab] OR “Nonalcoholic Steatohepatitides”[tiab] OR “Steatohepatitides, Nonalcoholic”[tiab] OR “Steatohepatitis, Nonalcoholic”[tiab] OR “MAFLD”[tiab] OR “Metabolic dysfunction-Associated Fatty Liver Disease”[tiab] OR “Metabolic dysfunction associated Fatty Liver Disease”[tiab] OR “Metabolic dysfunction-associated steatotic liver disease*”[tiab] OR “Metabolic dysfunction associated steatotic liver disease*”[tiab] OR “MASLD”[tiab]) | 50 |
| **Embase** | (“Triglyceride Glucose-BMI”:ti,ab,kw OR “Triglyceride Glucose-BMI”:ti,ab,kw OR “TyG-BMI”:ti,ab,kw OR “Triglyceride Glucose-Body Mass Index”:ti,ab,kw OR “Triglyceride Glucose Body Mass Index”:ti,ab,kw OR “TyG-Body Mass Index”:ti,ab,kw OR “TyG-BMI Index”:ti,ab,kw)  AND  (“Non-alcoholic Fatty Liver Disease”/exp OR “Non-alcoholic Fatty Liver Disease”:ti,ab,kw OR “Non alcoholic Fatty Liver Disease”:ti,ab,kw OR “NAFLD”:ti,ab,kw OR “Nonalcoholic Fatty Liver Disease”:ti,ab,kw OR “Fatty Liver, Nonalcoholic”:ti,ab,kw OR “Fatty Livers, Nonalcoholic”:ti,ab,kw OR “Liver, Nonalcoholic Fatty”:ti,ab,kw OR “Livers, Nonalcoholic Fatty”:ti,ab,kw OR “Nonalcoholic Fatty Liver”:ti,ab,kw OR “Nonalcoholic Fatty Livers”:ti,ab,kw OR “Nonalcoholic Steatohepatitis”:ti,ab,kw OR “Nonalcoholic Steatohepatitides”:ti,ab,kw OR “Steatohepatitides, Nonalcoholic”:ti,ab,kw OR “Steatohepatitis, Nonalcoholic”:ti,ab,kw OR “MAFLD”:ti,ab,kw OR “Metabolic dysfunction-Associated Fatty Liver Disease”:ti,ab,kw OR “Metabolic dysfunction associated Fatty Liver Disease”:ti,ab,kw OR “MASLD”:ti,ab,kw OR “Metabolic dysfunction-associated steatotic liver disease*”:ti,ab,kw OR “Metabolic dysfunction associated steatotic liver disease*”:ti,ab,kw) | 57 |
| **Scopus** | TITLE-ABS-KEY(“Triglyceride Glucose-BMI” OR “TyG-BMI” OR “Triglyceride Glucose-Body Mass Index” OR “Triglyceride Glucose Body Mass Index” OR “TyG-Body Mass Index” OR “TyG-BMI Index”)  AND  TITLE-ABS-KEY(“Non-alcoholic Fatty Liver Disease” OR “Non alcoholic Fatty Liver Disease” OR “NAFLD” OR “Nonalcoholic Fatty Liver Disease” OR “Fatty Liver, Nonalcoholic” OR “Fatty Livers, Nonalcoholic” OR “Liver, Nonalcoholic Fatty” OR “Livers, Nonalcoholic Fatty” OR “Nonalcoholic Fatty Liver” OR “Nonalcoholic Fatty Livers” OR “Nonalcoholic Steatohepatitis” OR “Nonalcoholic Steatohepatitides” OR “Steatohepatitides, Nonalcoholic” OR “Steatohepatitis, Nonalcoholic” OR “Metabolic dysfunction-Associated Fatty Liver Disease” OR “MAFLD” OR “Metabolic dysfunction associated Fatty Liver Disease” OR “MASLD” OR “Metabolic dysfunction-associated steatotic liver disease*” OR “Metabolic dysfunction associated steatotic liver disease*”) | 50 |
| **Web of Science** | TS=(“Triglyceride Glucose-BMI” OR “TyG-BMI” OR “Triglyceride Glucose-Body Mass Index” OR “Triglyceride Glucose Body Mass Index” OR “TyG-Body Mass Index” OR “TyG-BMI Index”)  AND  TS=(“Non-alcoholic Fatty Liver Disease” OR “Non alcoholic Fatty Liver Disease” OR “NAFLD” OR “Nonalcoholic Fatty Liver Disease” OR “Fatty Liver, Nonalcoholic” OR “Fatty Livers, Nonalcoholic” OR “Liver, Nonalcoholic Fatty” OR “Livers, Nonalcoholic Fatty” OR “Nonalcoholic Fatty Liver” OR “Nonalcoholic Fatty Livers” OR “Nonalcoholic Steatohepatitis” OR “Nonalcoholic Steatohepatitides” OR “Steatohepatitides, Nonalcoholic” OR “Steatohepatitis, Nonalcoholic” OR “Metabolic dysfunction-Associated Fatty Liver Disease” OR “MAFLD” OR “Metabolic dysfunction associated Fatty Liver Disease” OR “MASLD” OR “Metabolic dysfunction-associated steatotic liver disease*” OR “Metabolic dysfunction associated steatotic liver disease*”) | 47 |
| **Total** | | **204** |
