# Supplementary material for: Association of triglyceride glucose-body mass index (TyG-BMI) with metabolic dysfunction-associated steatotic liver disease: A systematic review and meta-analysis
Source: PLoS One. 2025 Aug 4;20(8):e0324483. doi: 10.1371/journal.pone.0324483 (PMC12321072; doi:10.1371/journal.pone.0324483)
Supplement: S2 Table — (DOCX) [file pone.0324483.s002.docx]

**Table S2.** List of all studies identified in literature search

| **Author and journal names and titles** |
| --- |
| 1. Bockarie AS, Nartey YA, Nsiah P, Edzie EKM, Tuoyire D, Acquah S, et al. Fatty liver biomarkers and insulin resistance indices in the prediction of non-alcoholic fatty liver disease in Ghanaian patients. Endocrinology, Diabetes and Metabolism. 2023;6(6).  2. Bockarie AS, Nartey YA, Nsiah P, Edzie EKM, Tuoyire D, Acquah S, et al. Fatty liver biomarkers and insulin resistance indices in the prediction of non-alcoholic fatty liver disease in Ghanaian patients. ENDOCRINOLOGY DIABETES & METABOLISM. 2023;6(6).  3. Bockarie AS, Nartey YA, Nsiah P, Edzie EKM, Tuoyire D, Acquah S, et al. Fatty liver biomarkers and insulin resistance indices in the prediction of non-alcoholic fatty liver disease in Ghanaian patients. Endocrinology, Diabetes and Metabolism. 2023;6(6).  4. Bockarie AS, Nartey YA, Nsiah P, Edzie EKM, Tuoyire D, Acquah S, et al. Fatty liver biomarkers and insulin resistance indices in the prediction of non-alcoholic fatty liver disease in Ghanaian patients. Endocrinol Diabetes Metab. 2023;6(6):e456.  5. Bullón-Vela V, Abete I, Zulet MA, Martínez JA. Association between triglyceride glucose-body mass index and risk factors linked to non-alcoholic liver disease in subjects with metabolic syndrome. Proceedings of the Nutrition Society. 2020;79(OCE2).  6. Chang M, Shao Z, Shen G. Association between triglyceride glucose-related markers and the risk of metabolic-associated fatty liver disease: a cross-sectional study in healthy Chinese participants. BMJ Open. 2023;13(5).  7. Chang M, Shao Z, Shen G. Association between triglyceride glucose-related markers and the risk of metabolic-associated fatty liver disease: a cross-sectional study in healthy Chinese participants. BMJ Open. 2023;13(5).  8. Chang M, Shao Z, Shen G. Association between triglyceride glucose-related markers and the risk of metabolic-associated fatty liver disease: a cross-sectional study in healthy Chinese participants. BMJ Open. 2023;13(5):e070189.  9. Chang MX, Shao ZH, Shen GF. Association between triglyceride glucose-related markers and the risk of metabolic-associated fatty liver disease: a cross-sectional study in healthy Chinese participants. BMJ OPEN. 2023;13(5).  10. Chen B, Peng XE. Association Between Serum Uric Acid to Creatinine Ratio and Metabolic-Associated Fatty Liver Disease in Southeast China (TyG-BMI as a Potential Mediator). Diabetes, Metabolic Syndrome and Obesity. 2024;17:4711-20.  11. Chen B, Peng XE. Association Between Serum Uric Acid to Creatinine Ratio and Metabolic-Associated Fatty Liver Disease in Southeast China (TyG-BMI as a Potential Mediator). Diabetes, Metabolic Syndrome and Obesity. 2024;17:4711-20.  12. Chen B, Peng XE. Association Between Serum Uric Acid to Creatinine Ratio and Metabolic-Associated Fatty Liver Disease in Southeast China (TyG-BMI as a Potential Mediator). Diabetes Metab Syndr Obes. 2024;17:4711-20.  13. Chen Q, Hu P, Hou X, Sun Y, Jiao M, Peng L, et al. Association between triglyceride-glucose related indices and mortality among individuals with non-alcoholic fatty liver disease or metabolic dysfunction-associated steatotic liver disease. Cardiovascular Diabetology. 2024;23(1).  14. Chen Q, Hu P, Hou X, Sun Y, Jiao M, Peng L, et al. Association between triglyceride-glucose related indices and mortality among individuals with non-alcoholic fatty liver disease or metabolic dysfunction-associated steatotic liver disease. Cardiovascular Diabetology. 2024;23(1).  15. Chen Q, Hu P, Hou X, Sun Y, Jiao M, Peng L, et al. Association between triglyceride-glucose related indices and mortality among individuals with non-alcoholic fatty liver disease or metabolic dysfunction-associated steatotic liver disease. Cardiovasc Diabetol. 2024;23(1):232.  16. Chen QL, Hu PP, Hou XX, Sun Y, Jiao MF, Peng LY, et al. Association between triglyceride-glucose related indices and mortality among individuals with non-alcoholic fatty liver disease or metabolic dysfunction-associated steatotic liver disease. CARDIOVASCULAR DIABETOLOGY. 2024;23(1).  17. Gao Q, Feng L, Zhou W, Li X, Yin L, Wang Y. Non-Traditional Blood Lipid Indices for Metabolism Dysfunction-Associated Fatty Liver Disease Prediction in Non-Obese Type 2 Diabetes Mellitus. Diabetes, Metabolic Syndrome and Obesity. 2022;16:2345-54.  18. Gao Q, Feng L, Zhou W, Li X, Yin L, Wang Y. Non-Traditional Blood Lipid Indices for Metabolism Dysfunction-Associated Fatty Liver Disease Prediction in Non-Obese Type 2 Diabetes Mellitus. Diabetes, Metabolic Syndrome and Obesity. 2023;16:2345-54.  19. Gao Q, Feng L, Zhou W, Li X, Yin L, Wang Y. Non-Traditional Blood Lipid Indices for Metabolism Dysfunction-Associated Fatty Liver Disease Prediction in Non-Obese Type 2 Diabetes Mellitus. Diabetes Metab Syndr Obes. 2023;16:2345-54.  20. Gao Q, Feng L, Zhou WL, Li XL, Yin LZ, Wang Y. Non-Traditional Blood Lipid Indices for Metabolism Dysfunction-Associated Fatty Liver Disease Prediction in Non-Obese Type 2 Diabetes Mellitus. DIABETES METABOLIC SYNDROME AND OBESITY. 2023;16:2345-54.  21. Han AL, Lee HK, Shin SR. Diagnostic Performance of Insulin Resistance Indices for Identifying Metabolic Dysfunction-Associated Fatty Liver Disease. Metabolic Syndrome and Related Disorders. 2024;22(5):402-9.  22. Han AL, Lee HK, Shin SR. Diagnostic Performance of Insulin Resistance Indices for Identifying Metabolic Dysfunction-Associated Fatty Liver Disease. METABOLIC SYNDROME AND RELATED DISORDERS. 2024;22(5):402-9.  23. Han AL, Lee HK, Shin SR. Diagnostic Performance of Insulin Resistance Indices for Identifying Metabolic Dysfunction-Associated Fatty Liver Disease. Metabolic Syndrome and Related Disorders. 2024;22(5):402-9.  24. Han AL, Lee HK, Shin SR. Diagnostic Performance of Insulin Resistance Indices for Identifying Metabolic Dysfunction-Associated Fatty Liver Disease. Metab Syndr Relat Disord. 2024;22(5):402-9.  25. Hosseini SA, Alipour M, Sarvandian S, Haghighat N, Bazyar H, Aghakhani L. Assessment of the appropriate cutoff points for anthropometric indices and their relationship with cardio-metabolic indices to predict the risk of metabolic associated fatty liver disease. BMC Endocrine Disorders. 2024;24(1).  26. Hosseini SA, Alipour M, Sarvandian S, Haghighat N, Bazyar H, Aghakhani L. Assessment of the appropriate cutoff points for anthropometric indices and their relationship with cardio-metabolic indices to predict the risk of metabolic associated fatty liver disease. BMC Endocrine Disorders. 2024;24(1).  27. Hosseini SA, Alipour M, Sarvandian S, Haghighat N, Bazyar H, Aghakhani L. Assessment of the appropriate cutoff points for anthropometric indices and their relationship with cardio-metabolic indices to predict the risk of metabolic associated fatty liver disease. BMC Endocr Disord. 2024;24(1):79.  28. Hu H, Han Y, Cao C, He Y. The triglyceride glucose-body mass index: a non-invasive index that identifies non-alcoholic fatty liver disease in the general Japanese population. Journal of Translational Medicine. 2022;20(1).  29. Hu H, Han Y, Cao C, He Y. The triglyceride glucose-body mass index: a non-invasive index that identifies non-alcoholic fatty liver disease in the general Japanese population. Journal of Translational Medicine. 2022;20(1).  30. Hu H, Han Y, Cao C, He Y. The triglyceride glucose-body mass index: a non-invasive index that identifies non-alcoholic fatty liver disease in the general Japanese population. J Transl Med. 2022;20(1):398.  31. Hu HF, Han Y, Cao CC, He YC. The triglyceride glucose-body mass index: a non-invasive index that identifies non-alcoholic fatty liver disease in the general Japanese population. JOURNAL OF TRANSLATIONAL MEDICINE. 2022;20(1).  32. Hu M, Yang J, Gao B, Wu Z, Wu Y, Hu D, et al. Prediction of MASLD using different screening indexes in Chinese type 2 diabetes mellitus. Diabetol Metab Syndr. 2025;17(1):10.  33. Hu MM, Yang JY, Gao BB, Wu ZL, Wu Y, Hu DD, et al. Prediction of MASLD using different screening indexes in Chinese type 2 diabetes mellitus. DIABETOLOGY & METABOLIC SYNDROME. 2025;17(1).  34. Huang D, Zhong X, Lu J, Shi Y. Insulin resistance is associated with multiple comorbidities of psoriasis and significantly reduces the efficacy of biologics in patients with psoriasis: An analysis from SPEECH in China. British Journal of Dermatology. 2024;191:iii18-iii9.  35. Hwang IT, Kim EY. The association of triglyceride glucose-body mass index with transient elastography in pediatric non-alcoholic fatty liver disease. Hormone Research in Paediatrics. 2023;96:215-6.  36. Hwang IT, Kim EY. The association of triglyceride glucose-body mass index with transient elastography in pediatric non-alcoholic fatty liver disease. HORMONE RESEARCH IN PAEDIATRICS. 2023;96:215-6.  37. Khamseh ME, Malek M, Abbasi R, Taheri H, Lahouti M, Alaei-Shahmiri F. Triglyceride Glucose Index and Related Parameters (Triglyceride Glucose-Body Mass Index and Triglyceride Glucose-Waist Circumference) Identify Nonalcoholic Fatty Liver and Liver Fibrosis in Individuals with Overweight/Obesity. Metabolic Syndrome and Related Disorders. 2021;19(3):167-73.  38. Khamseh ME, Malek M, Abbasi R, Taheri H, Lahouti M, Alaei-Shahmiri F. Triglyceride Glucose Index and Related Parameters (Triglyceride Glucose-Body Mass Index and Triglyceride Glucose-Waist Circumference) Identify Nonalcoholic Fatty Liver and Liver Fibrosis in Individuals with Overweight/Obesity. METABOLIC SYNDROME AND RELATED DISORDERS. 2021;19(3):167-73.  39. Khamseh ME, Malek M, Abbasi R, Taheri H, Lahouti M, Alaei-Shahmiri F. Triglyceride Glucose Index and Related Parameters (Triglyceride Glucose-Body Mass Index and Triglyceride Glucose-Waist Circumference) Identify Nonalcoholic Fatty Liver and Liver Fibrosis in Individuals with Overweight/Obesity. Metabolic Syndrome and Related Disorders. 2021;19(3):167-73.  40. Khamseh ME, Malek M, Abbasi R, Taheri H, Lahouti M, Alaei-Shahmiri F. Triglyceride Glucose Index and Related Parameters (Triglyceride Glucose-Body Mass Index and Triglyceride Glucose-Waist Circumference) Identify Nonalcoholic Fatty Liver and Liver Fibrosis in Individuals with Overweight/Obesity. Metab Syndr Relat Disord. 2021;19(3):167-73.  41. Khamseh ME, Malek M, Jahangiri S, Nobarani S, Hekmatdoost A, Salavatizadeh M, et al. Insulin Resistance/Sensitivity Measures as Screening Indicators of Metabolic-Associated Fatty Liver Disease and Liver Fibrosis. Digestive Diseases and Sciences. 2024;69(4):1430-43.  42. Khamseh ME, Malek M, Jahangiri S, Nobarani S, Hekmatdoost A, Salavatizadeh M, et al. Insulin Resistance/Sensitivity Measures as Screening Indicators of Metabolic-Associated Fatty Liver Disease and Liver Fibrosis. DIGESTIVE DISEASES AND SCIENCES. 2024;69(4):1430-43.  43. Khamseh ME, Malek M, Jahangiri S, Nobarani S, Hekmatdoost A, Salavatizadeh M, et al. Insulin Resistance/Sensitivity Measures as Screening Indicators of Metabolic-Associated Fatty Liver Disease and Liver Fibrosis. Digestive Diseases and Sciences. 2024;69(4):1430-43.  44. Khamseh ME, Malek M, Jahangiri S, Nobarani S, Hekmatdoost A, Salavatizadeh M, et al. Insulin Resistance/Sensitivity Measures as Screening Indicators of Metabolic-Associated Fatty Liver Disease and Liver Fibrosis. Dig Dis Sci. 2024;69(4):1430-43.  45. Kilonzo SB, Kamala E, Jaka H, Ngoya P. Non-alcoholic fatty liver disease in Tanzania: prevalence, determinants, and diagnostic performance of triglycerides-glucose index and triglycerides-glucose index –body mass index compared to the hepatic ultrasound in overweight and obese individuals. BMC Gastroenterology. 2024;24(1).  46. Kilonzo SB, Kamala E, Jaka H, Ngoya P. Non-alcoholic fatty liver disease in Tanzania: prevalence, determinants, and diagnostic performance of triglycerides-glucose index and triglycerides-glucose index -body mass index compared to the hepatic ultrasound in overweight and obese individuals. BMC GASTROENTEROLOGY. 2024;24(1).  47. Kilonzo SB, Kamala E, Jaka H, Ngoya P. Non-alcoholic fatty liver disease in Tanzania: prevalence, determinants, and diagnostic performance of triglycerides-glucose index and triglycerides-glucose index –body mass index compared to the hepatic ultrasound in overweight and obese individuals. BMC Gastroenterology. 2024;24(1).  48. Kilonzo SB, Kamala E, Jaka H, Ngoya P. Non-alcoholic fatty liver disease in Tanzania: prevalence, determinants, and diagnostic performance of triglycerides-glucose index and triglycerides-glucose index -body mass index compared to the hepatic ultrasound in overweight and obese individuals. BMC Gastroenterol. 2024;24(1):96.  49. Kim AH, Son DH, Lee YJ. Modified triglyceride-glucose index indices are reliable markers for predicting risk of metabolic dysfunction-associated fatty liver disease: a cross-sectional study. Frontiers in Endocrinology. 2023;14.  50. Kim AH, Son DH, Lee YJ. Modified triglyceride-glucose index indices are reliable markers for predicting risk of metabolic dysfunction-associated fatty liver disease: a cross-sectional study. Frontiers in Endocrinology. 2023;14.  51. Kim AH, Son DH, Lee YJ. Modified triglyceride-glucose index indices are reliable markers for predicting risk of metabolic dysfunction-associated fatty liver disease: a cross-sectional study. Front Endocrinol (Lausanne). 2023;14:1308265.  52. Kim AH, Son DH, Lee YJ. Modified triglyceride-glucose index indices are reliable markers for predicting risk of metabolic dysfunction-associated fatty liver disease: a cross-sectional study. FRONTIERS IN ENDOCRINOLOGY. 2024;14.  53. Kim HS, Cho YK, Kim EH, Lee MJ, Jung CH, Park JY, et al. Triglyceride Glucose-Waist Circumference Is Superior to the Homeostasis Model Assessment of Insulin Resistance in Identifying Nonalcoholic Fatty Liver Disease in Healthy Subjects. J Clin Med. 2021;11(1).  54. Kim HS, Cho YK, Kim EH, Lee MJ, Jung CH, Park JY, et al. Triglyceride glucose-waist circumference is superior to the homeostasis model assessment of insulin resistance in identifying nonalcoholic fatty liver disease in healthy subjects. Journal of Clinical Medicine. 2022;11(1).  55. Kim HS, Cho YK, Kim EH, Lee MJ, Jung CH, Park JY, et al. Triglyceride Glucose-Waist Circumference Is Superior to the Homeostasis Model Assessment of Insulin Resistance in Identifying Nonalcoholic Fatty Liver Disease in Healthy Subjects. JOURNAL OF CLINICAL MEDICINE. 2022;11(1).  56. Kim HS, Cho YK, Kim EH, Lee MJ, Jung CH, Park JY, et al. Triglyceride glucose-waist circumference is superior to the homeostasis model assessment of insulin resistance in identifying nonalcoholic fatty liver disease in healthy subjects. Journal of Clinical Medicine. 2022;11(1).  57. Kuang M, Yang R, Huang X, Wang C, Sheng G, Xie G, et al. Assessing temporal differences in the predictive power of baseline TyG-related parameters for future diabetes: an analysis using time-dependent receiver operating characteristics. Journal of Translational Medicine. 2023;21(1).  58. Kuang M, Yang R, Huang X, Wang C, Sheng G, Xie G, et al. Assessing temporal differences in the predictive power of baseline TyG-related parameters for future diabetes: an analysis using time-dependent receiver operating characteristics. Journal of Translational Medicine. 2023;21(1).  59. Lee J, Han CI, Lee DY, Sung PS, Bae SH, Yang H. Performance of Noninvasive Indices for Discrimination of Metabolic Dysfunction-Associated Steatotic Liver Disease in Young Adults. Gut and liver. 2024.  60. Lee J, Han CI, Lee DY, Sung PS, Bae SH, Yang H. Performance of Noninvasive Indices for Discrimination of Metabolic Dysfunction-Associated Steatotic Liver Disease in Young Adults. Gut and Liver. 2025;19(1):116-25.  61. Lee J, Han CI, Lee DY, Sung PS, Bae SH, Yang H. Performance of Noninvasive Indices for Discrimination of Metabolic Dysfunction-Associated Steatotic Liver Disease in Young Adults. Gut Liver. 2025;19(1):116-25.  62. Lee J, Yang H. Performance of Non-Invasive Steatosis Indices for Discrimination of Metabolic Dysfunction-Associated Steatotic Liver Disease in Young Adults. Gut and Liver. 2024;18:106.  63. Li D, Wu S, Zhang M, Xie C, Tan H, Li N. Correlation between triglyceride-glucose index and related parameters and nonalcoholic fatty liver disease in northwest China. International Journal of Diabetes in Developing Countries. 2024;44(3):496-502.  64. Li D, Wu S, Zhang M, Xie C, Tan H, Li N. Correlation between triglyceride-glucose index and related parameters and nonalcoholic fatty liver disease in northwest China. International Journal of Diabetes in Developing Countries. 2024;44(3):496-502.  65. Li DT, Wu SL, Zhang MY, Xie CY, Tan HW, Li N. Correlation between triglyceride-glucose index and related parameters and nonalcoholic fatty liver disease in northwest China. INTERNATIONAL JOURNAL OF DIABETES IN DEVELOPING COUNTRIES. 2024;44(3):496-502.  66. Li H, Gao G, Xu Z, Zhao L, Xing Y, He J, et al. Association and Diagnostic Value of TyG-BMI for Hyperuricemia in Patients with Non-Alcoholic Fatty Liver Disease: A Cross-Sectional Study. Diabetes, Metabolic Syndrome and Obesity. 2024;17:4663-73.  67. Li H, Gao G, Xu Z, Zhao L, Xing Y, He J, et al. Association and Diagnostic Value of TyG-BMI for Hyperuricemia in Patients with Non-Alcoholic Fatty Liver Disease: A Cross-Sectional Study. Diabetes, Metabolic Syndrome and Obesity. 2024;17:4663-73.  68. Li H, Gao G, Xu Z, Zhao L, Xing Y, He J, et al. Association and Diagnostic Value of TyG-BMI for Hyperuricemia in Patients with Non-Alcoholic Fatty Liver Disease: A Cross-Sectional Study. Diabetes Metab Syndr Obes. 2024;17:4663-73.  69. Li H, Shi Z, Chen X, Wang J, Ding J, Geng S, et al. Relationship Between Six Insulin Resistance Surrogates and Nonalcoholic Fatty Liver Disease Among Older Adults: A Cross-Sectional Study. Diabetes, Metabolic Syndrome and Obesity. 2023;16:1685-96.  70. Li H, Shi Z, Chen X, Wang J, Ding J, Geng S, et al. Relationship Between Six Insulin Resistance Surrogates and Nonalcoholic Fatty Liver Disease Among Older Adults: A Cross-Sectional Study. Diabetes, Metabolic Syndrome and Obesity. 2023;16:1685-96.  71. Li H, Shi Z, Chen X, Wang J, Ding J, Geng S, et al. Relationship Between Six Insulin Resistance Surrogates and Nonalcoholic Fatty Liver Disease Among Older Adults: A Cross-Sectional Study. Diabetes Metab Syndr Obes. 2023;16:1685-96.  72. Li HJ, Shi Z, Chen XJ, Wang JJ, Ding JC, Geng SJ, et al. Relationship Between Six Insulin Resistance Surrogates and Nonalcoholic Fatty Liver Disease Among Older Adults: A Cross-Sectional Study. DIABETES METABOLIC SYNDROME AND OBESITY. 2023;16:1685-96.  73. Li N, Tan H, Xie A, Li C, Fu X, Xang W, et al. Value of the triglyceride glucose index combined with body mass index in identifying non-alcoholic fatty liver disease in patients with type 2 diabetes. BMC Endocrine Disorders. 2022;22(1).  74. Li N, Tan H, Xie A, Li C, Fu X, Xang W, et al. Value of the triglyceride glucose index combined with body mass index in identifying non-alcoholic fatty liver disease in patients with type 2 diabetes. BMC Endocrine Disorders. 2022;22(1).  75. Li N, Tan H, Xie A, Li C, Fu X, Xang W, et al. Value of the triglyceride glucose index combined with body mass index in identifying non-alcoholic fatty liver disease in patients with type 2 diabetes. BMC Endocr Disord. 2022;22(1):101.  76. Li N, Tan HW, Xie AX, Li C, Fu X, Xang WT, et al. Value of the triglyceride glucose index combined with body mass index in identifying non-alcoholic fatty liver disease in patients with type 2 diabetes. BMC ENDOCRINE DISORDERS. 2022;22(1).  77. Li S, Feng L, Ding J, Zhou W, Yuan T, Mao J. Triglyceride glucose-waist circumference: the optimum index to screen nonalcoholic fatty liver disease in non-obese adults. BMC Gastroenterology. 2023;23(1).  78. Li S, Feng L, Ding J, Zhou W, Yuan T, Mao J. Triglyceride glucose-waist circumference: the optimum index to screen nonalcoholic fatty liver disease in non-obese adults. BMC Gastroenterology. 2023;23(1).  79. Li S, Feng L, Ding J, Zhou W, Yuan T, Mao J. Triglyceride glucose-waist circumference: the optimum index to screen nonalcoholic fatty liver disease in non-obese adults. BMC Gastroenterol. 2023;23(1):376.  80. Li SY, Feng L, Ding J, Zhou WH, Yuan TB, Mao JF. Triglyceride glucose-waist circumference: the optimum index to screen nonalcoholic fatty liver disease in non-obese adults. BMC GASTROENTEROLOGY. 2023;23(1).  81. Li Y, Zheng R, Li J, Feng S, Wang L, Huang Z. Association between triglyceride glucose-body mass index and non-alcoholic fatty liver disease in the non-obese Chinese population with normal blood lipid levels: a secondary analysis based on a prospective cohort study. Lipids in Health and Disease. 2020;19(1).  82. Li Y, Zheng R, Li J, Feng S, Wang L, Huang Z. Association between triglyceride glucose-body mass index and non-alcoholic fatty liver disease in the non-obese Chinese population with normal blood lipid levels: a secondary analysis based on a prospective cohort study. Lipids in Health and Disease. 2020;19(1).  83. Li Y, Zheng R, Li J, Feng S, Wang L, Huang Z. Association between triglyceride glucose-body mass index and non-alcoholic fatty liver disease in the non-obese Chinese population with normal blood lipid levels: a secondary analysis based on a prospective cohort study. Lipids Health Dis. 2020;19(1):229.  84. Li YL, Zheng R, Li J, Feng SY, Wang L, Huang ZM. Association between triglyceride glucose-body mass index and non-alcoholic fatty liver disease in the non-obese Chinese population with normal blood lipid levels: a secondary analysis based on a prospective cohort study. LIPIDS IN HEALTH AND DISEASE. 2020;19(1).  85. Lim J. Validation of fatty liver index in a healthy Korean population and its comparison with triglyceride glucose index and its related parameters. Clinica Chimica Acta. 2019;493:S374.  86. Liu Z, He H, Dai Y, Yang L, Liao S, An Z, et al. Comparison of the diagnostic value between triglyceride-glucose index and triglyceride to high-density lipoprotein cholesterol ratio in metabolic-associated fatty liver disease patients: a retrospective cross-sectional study. Lipids in Health and Disease. 2022;21(1).  87. Liu Z, He H, Dai Y, Yang L, Liao S, An Z, et al. Comparison of the diagnostic value between triglyceride-glucose index and triglyceride to high-density lipoprotein cholesterol ratio in metabolic-associated fatty liver disease patients: a retrospective cross-sectional study. Lipids in Health and Disease. 2022;21(1).  88. Liu Z, He H, Dai Y, Yang L, Liao S, An Z, et al. Comparison of the diagnostic value between triglyceride-glucose index and triglyceride to high-density lipoprotein cholesterol ratio in metabolic-associated fatty liver disease patients: a retrospective cross-sectional study. Lipids Health Dis. 2022;21(1):55.  89. Liu Z, He H, Dai YZ, Yang LD, Liao SL, An ZM, et al. Comparison of the diagnostic value between triglyceride-glucose index and triglyceride to high-density lipoprotein cholesterol ratio in metabolic-associated fatty liver disease patients: a retrospective cross-sectional study. LIPIDS IN HEALTH AND DISEASE. 2022;21(1).  90. Malek M, Khamseh ME, Chehrehgosha H, Nobarani S, Alaei-Shahmiri F. Triglyceride glucose-waist to height ratio: a novel and effective marker for identifying hepatic steatosis in individuals with type 2 diabetes mellitus. Endocrine. 2021;74(3):538-45.  91. Malek M, Khamseh ME, Chehrehgosha H, Nobarani S, Alaei-Shahmiri F. Triglyceride glucose-waist to height ratio: a novel and effective marker for identifying hepatic steatosis in individuals with type 2 diabetes mellitus. ENDOCRINE. 2021;74(3):538-45.  92. Malek M, Khamseh ME, Chehrehgosha H, Nobarani S, Alaei-Shahmiri F. Triglyceride glucose-waist to height ratio: a novel and effective marker for identifying hepatic steatosis in individuals with type 2 diabetes mellitus. Endocrine. 2021;74(3):538-45.  93. Malek M, Khamseh ME, Chehrehgosha H, Nobarani S, Alaei-Shahmiri F. Triglyceride glucose-waist to height ratio: a novel and effective marker for identifying hepatic steatosis in individuals with type 2 diabetes mellitus. Endocrine. 2021;74(3):538-45.  94. Milošević N, Milanović M, Sudji J, Bosić Živanović D, Stojanoski S, Vuković B, et al. Could phthalates exposure contribute to the development of metabolic syndrome and liver disease in humans? Environmental science and pollution research international. 2020;27(1):772-84.  95. Milošević N, Milanović M, Sudji J, Bosić Živanović D, Stojanoski S, Vuković B, et al. Could phthalates exposure contribute to the development of metabolic syndrome and liver disease in humans? Environmental Science and Pollution Research. 2020;27(1):772-84.  96. Milošević N, Milanović M, Sudji J, Bosić Živanović D, Stojanoski S, Vuković B, et al. Could phthalates exposure contribute to the development of metabolic syndrome and liver disease in humans? Environ Sci Pollut Res Int. 2020;27(1):772-84.  97. Milosevic N, Milanovic M, Sudji J, Zivanovic DB, Stojanoski S, Vukovic B, et al. Could phthalates exposure contribute to the development of metabolic syndrome and liver disease in humans? ENVIRONMENTAL SCIENCE AND POLLUTION RESEARCH. 2020;27(1):772-84.  98. Otsubo N, Fukuda T, Cho G, Ishibashi F, Yamada T, Monzen K. Utility of Indices Obtained during Medical Checkups for Predicting Fatty Liver Disease in Non-obese People. Internal Medicine. 2023;62(16):2307-19.  99. Otsubo N, Fukuda T, Cho G, Ishibashi F, Yamada T, Monzen K. Utility of Indices Obtained during Medical Checkups for Predicting Fatty Liver Disease in Non-obese People. Internal Medicine. 2023;62(16):2307-19.  100. Otsubo N, Fukuda T, Cho G, Ishibashi F, Yamada T, Monzen K. Utility of Indices Obtained during Medical Checkups for Predicting Fatty Liver Disease in Non-obese People. Intern Med. 2023;62(16):2307-19.  101. Otsubo N, Fukuda T, Cho GH, Ishibashi F, Yamada T, Monzen K. Utility of Indices Obtained during Medical Checkups for Predicting Fatty Liver Disease in Non-obese People. INTERNAL MEDICINE. 2023;62(16):2307-19.  102. Peng H, Pan L, Ran S, Wang M, Huang S, Zhao M, et al. Prediction of MAFLD and NAFLD using different screening indexes: A cross-sectional study in U.S. adults. Frontiers in Endocrinology. 2023;14.  103. Peng H, Pan L, Ran S, Wang M, Huang S, Zhao M, et al. Prediction of MAFLD and NAFLD using different screening indexes: A cross-sectional study in U.S. adults. Frontiers in Endocrinology. 2023;14.  104. Peng H, Pan L, Ran S, Wang M, Huang S, Zhao M, et al. Prediction of MAFLD and NAFLD using different screening indexes: A cross-sectional study in U.S. adults. Front Endocrinol (Lausanne). 2023;14:1083032.  105. Peng HY, Pan L, Ran SM, Wang MY, Huang SX, Zhao M, et al. Prediction of MAFLD and NAFLD using different screening indexes: A cross-sectional study in US adults. FRONTIERS IN ENDOCRINOLOGY. 2023;14.  106. Peng N, Kuang M, Peng Y, Yu H, Zhang S, Xie G, et al. Associations between TyG-BMI and normal-high blood pressure values and hypertension: cross-sectional evidence from a non-diabetic population. Frontiers in Cardiovascular Medicine. 2023;10.  107. Peng N, Kuang M, Peng Y, Yu H, Zhang S, Xie G, et al. Associations between TyG-BMI and normal-high blood pressure values and hypertension: cross-sectional evidence from a non-diabetic population. Frontiers in Cardiovascular Medicine. 2023;10.  108. Peng N, Kuang M, Peng Y, Yu H, Zhang S, Xie G, et al. Associations between TyG-BMI and normal-high blood pressure values and hypertension: cross-sectional evidence from a non-diabetic population. Front Cardiovasc Med. 2023;10:1129112.  109. Peng N, Kuang MB, Peng Y, Yu H, Zhang SH, Xie GB, et al. Associations between TyG-BMI and normal-high blood pressure values and hypertension: cross-sectional evidence from a non-diabetic population. FRONTIERS IN CARDIOVASCULAR MEDICINE. 2023;10.  110. Półkośnik K, Bobrus-Chociej A, Czajkowska A, Lebensztejn D. COMPARISON OF SEVERAL POTENTIAL INDICES OF INSULIN RESISTANCE IN PREDICTING METABOLIC DYSFUNCTION-ASSOCIATED FATTY LIVER DISEASE IN OBESE CHILDREN. Journal of Pediatric Gastroenterology and Nutrition. 2023;76:881.  111. Priego-Parra BA, Reyes-Diaz SA, Ordaz-Alvarez HR, Bernal-Reyes R, Icaza-Chávez ME, Martínez-Vázquez SE, et al. Diagnostic performance of sixteen biomarkers for MASLD: A study in a Mexican cohort. CLINICS AND RESEARCH IN HEPATOLOGY AND GASTROENTEROLOGY. 2024;48(7).  112. Priego-Parra BA, Reyes-Diaz SA, Ordaz-Alvarez HR, Bernal-Reyes R, Icaza-Chávez ME, Martínez-Vázquez SE, et al. Diagnostic performance of sixteen biomarkers for MASLD: A study in a Mexican cohort. Clin Res Hepatol Gastroenterol. 2024;48(7):102400.  113. Priego-Parra BA, Reyes-Diaz SA, Ordaz-Alvarez HR, Bernal-Reyes R, Icaza-Chávez ME, Martínez-Vázquez SE, et al. Diagnostic performance of sixteen biomarkers for MASLD: A study in a Mexican cohort. Clinics and Research in Hepatology and Gastroenterology. 2024;48(7).  114. Priego-Parra BA, Reyes-Diaz SA, Ordaz-Alvarez HR, Bernal-Reyes R, Icaza-Chávez ME, Martínez-Vázquez SE, et al. Diagnostic performance of sixteen biomarkers for MASLD: A study in a Mexican cohort. Clinics and Research in Hepatology and Gastroenterology. 2024;48(7).  115. Qiao Y, Wang Y, Chen C, Huang Y, Zhao C. Association between triglyceride-glucose (TyG) related indices and cardiovascular diseases and mortality among individuals with metabolic dysfunction-associated steatotic liver disease: a cohort study of UK Biobank. Cardiovasc Diabetol. 2025;24(1):12.  116. Sheng G, Lu S, Xie Q, Peng N, Kuang M, Zou Y. The usefulness of obesity and lipid-related indices to predict the presence of Non-alcoholic fatty liver disease. Lipids in Health and Disease. 2021;20(1).  117. Sheng G, Lu S, Xie Q, Peng N, Kuang M, Zou Y. The usefulness of obesity and lipid-related indices to predict the presence of Non-alcoholic fatty liver disease. Lipids in Health and Disease. 2021;20(1).  118. Sheng G, Lu S, Xie Q, Peng N, Kuang M, Zou Y. The usefulness of obesity and lipid-related indices to predict the presence of Non-alcoholic fatty liver disease. Lipids Health Dis. 2021;20(1):134.  119. Sheng GT, Lu S, Xie QY, Peng N, Kuang MB, Zou Y. The usefulness of obesity and lipid-related indices to predict the presence of Non-alcoholic fatty liver disease. LIPIDS IN HEALTH AND DISEASE. 2021;20(1).  120. Song K, Park G, Lee HS, Lee M, Lee HI, Choi HS, et al. Comparison of the Triglyceride Glucose Index and Modified Triglyceride Glucose Indices to Predict Nonalcoholic Fatty Liver Disease in Youths. Journal of Pediatrics. 2022;242:79-85.e1.  121. Song K, Park G, Lee HS, Lee M, Lee HI, Choi HS, et al. Comparison of the Triglyceride Glucose Index and Modified Triglyceride Glucose Indices to Predict Nonalcoholic Fatty Liver Disease in Youths. JOURNAL OF PEDIATRICS. 2022;242:79-85.  122. Song K, Park G, Lee HS, Lee M, Lee HI, Choi HS, et al. Comparison of the Triglyceride Glucose Index and Modified Triglyceride Glucose Indices to Predict Nonalcoholic Fatty Liver Disease in Youths. Journal of Pediatrics. 2022;242:79-85.e1.  123. Song K, Park G, Lee HS, Lee M, Lee HI, Choi HS, et al. Comparison of the Triglyceride Glucose Index and Modified Triglyceride Glucose Indices to Predict Nonalcoholic Fatty Liver Disease in Youths. J Pediatr. 2022;242:79-85.e1.  124. Song S, Son DH, Baik SJ, Cho WJ, Lee YJ. Triglyceride Glucose-Waist Circumference (TyG-WC) Is a Reliable Marker to Predict Non-Alcoholic Fatty Liver Disease. BIOMEDICINES. 2022;10(9).  125. Song S, Son DH, Baik SJ, Cho WJ, Lee YJ. Triglyceride Glucose-Waist Circumference (TyG-WC) Is a Reliable Marker to Predict Non-Alcoholic Fatty Liver Disease. Biomedicines. 2022;10(9).  126. Song S, Son DH, Baik SJ, Cho WJ, Lee YJ. Triglyceride Glucose-Waist Circumference (TyG-WC) Is a Reliable Marker to Predict Non-Alcoholic Fatty Liver Disease. Biomedicines. 2022;10(9).  127. Tian J, Cao Y, Zhang W, Wang A, Yang X, Dong Y, et al. The potential of insulin resistance indices to predict non-alcoholic fatty liver disease in patients with type 2 diabetes. BMC Endocrine Disorders. 2024;24(1).  128. Tian J, Cao Y, Zhang W, Wang A, Yang X, Dong Y, et al. The potential of insulin resistance indices to predict non-alcoholic fatty liver disease in patients with type 2 diabetes. BMC Endocrine Disorders. 2024;24(1).  129. Tian J, Cao Y, Zhang W, Wang A, Yang X, Dong Y, et al. The potential of insulin resistance indices to predict non-alcoholic fatty liver disease in patients with type 2 diabetes. BMC Endocr Disord. 2024;24(1):261.  130. Tian J, Cao YT, Zhang WH, Wang AY, Yang XY, Dong YF, et al. The potential of insulin resistance indices to predict non-alcoholic fatty liver disease in patients with type 2 diabetes. BMC ENDOCRINE DISORDERS. 2024;24(1).  131. Trochimczyk K, Flisiak-Jackiewicz M, Bobrus-Chociej A, Lebensztejn A, Wojtkowska M, Jamiołkowski J, et al. Biochemical and Anthropometric Indices of Insulin Resistance in Obese and Overweight Children with Metabolic Dysfunction-Associated Fatty Liver Disease. Medical Science Monitor. 2024;30.  132. Trochimczyk K, Flisiak-Jackiewicz M, Bobrus-Chociej A, Lebensztejn A, Wojtkowska M, Jamiolkowski J, et al. Biochemical and Anthropometric Indices of Insulin Resistance in Obese and Overweight Children with Metabolic Dysfunction-Associated Fatty Liver Disease. MEDICAL SCIENCE MONITOR. 2024;30.  133. Trochimczyk K, Flisiak-Jackiewicz M, Bobrus-Chociej A, Lebensztejn A, Wojtkowska M, Jamiołkowski J, et al. Biochemical and Anthropometric Indices of Insulin Resistance in Obese and Overweight Children with Metabolic Dysfunction-Associated Fatty Liver Disease. Medical Science Monitor. 2024;30.  134. Trochimczyk K, Flisiak-Jackiewicz M, Bobrus-Chociej A, Lebensztejn A, Wojtkowska M, Jamiołkowski J, et al. Biochemical and Anthropometric Indices of Insulin Resistance in Obese and Overweight Children with Metabolic Dysfunction-Associated Fatty Liver Disease. Med Sci Monit. 2024;30:e943375.  135. Wang M, Chang M, Shen P, Wei W, Li H, Shen G. Application value of triglyceride-glucose index and triglyceride-glucose body mass index in evaluating the degree of hepatic steatosis in non-alcoholic fatty liver disease. Lipids in Health and Disease. 2023;22(1).  136. Wang M, Chang M, Shen P, Wei W, Li H, Shen G. Application value of triglyceride-glucose index and triglyceride-glucose body mass index in evaluating the degree of hepatic steatosis in non-alcoholic fatty liver disease. Lipids in Health and Disease. 2023;22(1).  137. Wang M, Chang M, Shen P, Wei W, Li H, Shen G. Application value of triglyceride-glucose index and triglyceride-glucose body mass index in evaluating the degree of hepatic steatosis in non-alcoholic fatty liver disease. Lipids Health Dis. 2023;22(1):186.  138. Wang MY, Chang MX, Shen PP, Wei W, Li HY, Shen GF. Application value of triglyceride-glucose index and triglyceride-glucose body mass index in evaluating the degree of hepatic steatosis in non-alcoholic fatty liver disease. LIPIDS IN HEALTH AND DISEASE. 2023;22(1).  139. Wang R, Dai L, Zhong Y, Xie G. Usefulness of the triglyceride glucose-body mass index in evaluating nonalcoholic fatty liver disease: insights from a general population. Lipids in Health and Disease. 2021;20(1).  140. Wang R, Dai L, Zhong Y, Xie G. Usefulness of the triglyceride glucose-body mass index in evaluating nonalcoholic fatty liver disease: insights from a general population. Lipids in Health and Disease. 2021;20(1).  141. Wang R, Dai L, Zhong Y, Xie G. Usefulness of the triglyceride glucose-body mass index in evaluating nonalcoholic fatty liver disease: insights from a general population. Lipids Health Dis. 2021;20(1):77.  142. Wang RS, Dai LL, Zhong YJ, Xie GB. Usefulness of the triglyceride glucose-body mass index in evaluating nonalcoholic fatty liver disease: insights from a general population. LIPIDS IN HEALTH AND DISEASE. 2021;20(1).  143. Wang X, Wang Z, Du W, Ma X, Ma J, Chen Z, et al. Predictive Value of TyG and TyG-BMI Indices for Non-Alcoholic Fatty Liver Disease in High-Altitude Regions of China: A Cross-Sectional Study. Journal of Clinical Medicine. 2024;13(23).  144. Wang X, Wang Z, Du W, Ma X, Ma J, Chen Z, et al. Predictive Value of TyG and TyG-BMI Indices for Non-Alcoholic Fatty Liver Disease in High-Altitude Regions of China: A Cross-Sectional Study. Journal of Clinical Medicine. 2024;13(23).  145. Wang X, Wang Z, Du W, Ma X, Ma J, Chen Z, et al. Predictive Value of TyG and TyG-BMI Indices for Non-Alcoholic Fatty Liver Disease in High-Altitude Regions of China: A Cross-Sectional Study. J Clin Med. 2024;13(23).  146. Wang XJ, Wang ZQ, Du W, Ma XB, Ma J, Chen ZJ, et al. Predictive Value of TyG and TyG-BMI Indices for Non-Alcoholic Fatty Liver Disease in High-Altitude Regions of China: A Cross-Sectional Study. JOURNAL OF CLINICAL MEDICINE. 2024;13(23).  147. Wang Y, Chen H. Clinical application of cluster analysis in patients with newly diagnosed type 2 diabetes. Hormones. 2024.  148. Wang Y, Chen H. Clinical application of cluster analysis in patients with newly diagnosed type 2 diabetes. Hormones. 2024.  149. Wang Y, Chen H. Clinical application of cluster analysis in patients with newly diagnosed type 2 diabetes. Hormones (Athens). 2024.  150. Wang YZ, Chen H. Clinical application of cluster analysis in patients with newly diagnosed type 2 diabetes. HORMONES-INTERNATIONAL JOURNAL OF ENDOCRINOLOGY AND METABOLISM. 2024.  151. Xiao Y, Wang H, Han L, Huang Z, Lyu G, Li S. Predictive value of anthropometric and biochemical indices in non-alcoholic fatty pancreas disease: a cross-sectional study. BMJ Open. 2024;14(4).  152. Xiao Y, Wang H, Han L, Huang Z, Lyu G, Li S. Predictive value of anthropometric and biochemical indices in non-alcoholic fatty pancreas disease: a cross-sectional study. BMJ Open. 2024;14(4).  153. Xiao Y, Wang H, Han L, Huang Z, Lyu G, Li S. Predictive value of anthropometric and biochemical indices in non-alcoholic fatty pancreas disease: a cross-sectional study. BMJ Open. 2024;14(4):e081131.  154. Xiao Y, Wang H, Han LN, Huang ZB, Lyu G, Li SL. Predictive value of anthropometric and biochemical indices in non-alcoholic fatty pancreas disease: a cross-sectional study. BMJ OPEN. 2024;14(4).  155. Xing Y, Chen J, Liu J, Ma H. Associations Between GGT/HDL and MAFLD: A Cross-Sectional Study. Diabetes, Metabolic Syndrome and Obesity. 2022;15:383-94.  156. Xing Y, Chen J, Liu J, Ma H. Associations Between GGT/HDL and MAFLD: A Cross-Sectional Study. Diabetes, Metabolic Syndrome and Obesity. 2022;15:383-94.  157. Xing Y, Chen J, Liu J, Ma H. Associations Between GGT/HDL and MAFLD: A Cross-Sectional Study. Diabetes Metab Syndr Obes. 2022;15:383-94.  158. Xing YL, Chen JH, Liu J, Ma HJ. Associations Between GGT/HDL and MAFLD: A Cross-Sectional Study. DIABETES METABOLIC SYNDROME AND OBESITY. 2022;15:383-94.  159. Xuan W, Liu D, Zhong J, Luo H, Zhang X. Impacts of Triglyceride Glucose-Waist to Height Ratio on Diabetes Incidence: A Secondary Analysis of A Population-Based Longitudinal Data. Frontiers in Endocrinology. 2022;13.  160. Xuan W, Liu D, Zhong J, Luo H, Zhang X. Impacts of Triglyceride Glucose-Waist to Height Ratio on Diabetes Incidence: A Secondary Analysis of A Population-Based Longitudinal Data. Frontiers in Endocrinology. 2022;13.  161. Xuan W, Liu D, Zhong J, Luo H, Zhang X. Impacts of Triglyceride Glucose-Waist to Height Ratio on Diabetes Incidence: A Secondary Analysis of A Population-Based Longitudinal Data. Front Endocrinol (Lausanne). 2022;13:949831.  162. Xuan WT, Liu DX, Zhong JA, Luo HJ, Zhang XW. Impacts of Triglyceride Glucose-Waist to Height Ratio on Diabetes Incidence: A Secondary Analysis of A Population-Based Longitudinal Data. FRONTIERS IN ENDOCRINOLOGY. 2022;13.  163. Xue Y, Xu J, Li M, Gao Y. Potential screening indicators for early diagnosis of NAFLD/MAFLD and liver fibrosis: Triglyceride glucose index–related parameters. Frontiers in Endocrinology. 2022;13.  164. Xue Y, Xu J, Li M, Gao Y. Potential screening indicators for early diagnosis of NAFLD/MAFLD and liver fibrosis: Triglyceride glucose index–related parameters. Frontiers in Endocrinology. 2022;13.  165. Xue Y, Xu J, Li M, Gao Y. Potential screening indicators for early diagnosis of NAFLD/MAFLD and liver fibrosis: Triglyceride glucose index-related parameters. Front Endocrinol (Lausanne). 2022;13:951689.  166. Xue Y, Xu JH, Li M, Gao YQ. Potential screening indicators for early diagnosis of NAFLD/MAFLD and liver fibrosis: Triglyceride glucose index-related parameters. FRONTIERS IN ENDOCRINOLOGY. 2022;13.  167. Yang X, Rao H, Yuan Y, Hu N, Zhang X, Zeng Y, et al. Correlation analysis of the triglyceride-glucose index and related parameters in metabolic dysfunction-associated fatty liver disease. Scientific reports. 2025;15(1):23.  168. Yang X, Rao H, Yuan Y, Hu N, Zhang X, Zeng Y, et al. Correlation analysis of the triglyceride–glucose index and related parameters in metabolic dysfunction-associated fatty liver disease. Scientific Reports. 2025;15(1).  169. Yang X, Rao H, Yuan Y, Hu N, Zhang X, Zeng Y, et al. Correlation analysis of the triglyceride-glucose index and related parameters in metabolic dysfunction-associated fatty liver disease. Sci Rep. 2025;15(1):23.  170. Yang X, Rao HT, Yuan Y, Hu N, Zhang XM, Zeng YX, et al. Correlation analysis of the triglyceride-glucose index and related parameters in metabolic dysfunction-associated fatty liver disease. SCIENTIFIC REPORTS. 2025;15(1).  171. Yang Z, Yu B, Wang Z, Li Z, Yang B, Zeng H, et al. Comparison of the prognostic value of a comprehensive set of predictors in identifying risk of metabolic-associated fatty liver disease among employed adults. BMC public health. 2023;23(1):584.  172. Yang Z, Yu B, Wang Z, Li Z, Yang B, Zeng H, et al. Comparison of the prognostic value of a comprehensive set of predictors in identifying risk of metabolic-associated fatty liver disease among employed adults. BMC Public Health. 2023;23(1).  173. Yang Z, Yu B, Wang Z, Li Z, Yang B, Zeng H, et al. Comparison of the prognostic value of a comprehensive set of predictors in identifying risk of metabolic-associated fatty liver disease among employed adults. BMC Public Health. 2023;23(1):584.  174. Yang Z, Yu B, Wang ZH, Li ZT, Yang B, Zeng HL, et al. Comparison of the prognostic value of a comprehensive set of predictors in identifying risk of metabolic-associated fatty liver disease among employed adults. BMC PUBLIC HEALTH. 2023;23(1).  175. Yoon JS, Kim BG, Hwang IT. Triglyceride-glucose-alanine aminotransferase index as a novel and superior predictor for non-alcoholic fatty liver disease in children and adolescents. Hormone Research in Paediatrics. 2022;95:221.  176. Yoon JS, Yi K. Triglyceride Glucose-body Mass Index Is A Reliable Surrogate Marker To Predict Non-alcoholic Fatty Liver Disease In Children And Adolescents. Journal of the Endocrine Society. 2023;7:A742-A3.  177. Zakerkish M, Hoseinian A, Alipour M, Payami SP. The Association between Cardio-metabolic and hepatic indices and anthropometric measures with metabolically obesity phenotypes: a cross-sectional study from the Hoveyzeh Cohort Study. BMC Endocrine Disorders. 2023;23(1).  178. Zakerkish M, Hoseinian A, Alipour M, Payami SP. The Association between Cardio-metabolic and hepatic indices and anthropometric measures with metabolically obesity phenotypes: a cross-sectional study from the Hoveyzeh Cohort Study. BMC ENDOCRINE DISORDERS. 2023;23(1).  179. Zakerkish M, Hoseinian A, Alipour M, Payami SP. The Association between Cardio-metabolic and hepatic indices and anthropometric measures with metabolically obesity phenotypes: a cross-sectional study from the Hoveyzeh Cohort Study. BMC Endocrine Disorders. 2023;23(1).  180. Zakerkish M, Hoseinian A, Alipour M, Payami SP. The Association between Cardio-metabolic and hepatic indices and anthropometric measures with metabolically obesity phenotypes: a cross-sectional study from the Hoveyzeh Cohort Study. BMC Endocr Disord. 2023;23(1):122.  181. Zeng P, Cai X, Yu X, Gong L. Markers of insulin resistance associated with non-alcoholic fatty liver disease in non-diabetic population. Scientific reports. 2023;13(1):20470.  182. Zeng P, Cai X, Yu X, Gong L. Markers of insulin resistance associated with non-alcoholic fatty liver disease in non-diabetic population. Scientific Reports. 2023;13(1).  183. Zeng P, Cai X, Yu X, Gong L. Markers of insulin resistance associated with non-alcoholic fatty liver disease in non-diabetic population. Sci Rep. 2023;13(1):20470.  184. Zeng P, Cai XS, Yu XZ, Gong LJ. Markers of insulin resistance associated with non-alcoholic fatty liver disease in non-diabetic population. SCIENTIFIC REPORTS. 2023;13(1).  185. Zhang F, Han Y, Wu Y, Bao Z, Zheng G, Liu J, et al. Association between triglyceride glucose-body mass index and the staging of non-alcoholic steatohepatitis and fibrosis in patients with non-alcoholic fatty liver disease. Annals of Medicine. 2024;56(1).  186. Zhang F, Han Y, Wu Y, Bao Z, Zheng G, Liu J, et al. Association between triglyceride glucose-body mass index and the staging of non-alcoholic steatohepatitis and fibrosis in patients with non-alcoholic fatty liver disease. Annals of Medicine. 2024;56(1).  187. Zhang F, Han Y, Wu Y, Bao Z, Zheng G, Liu J, et al. Association between triglyceride glucose-body mass index and the staging of non-alcoholic steatohepatitis and fibrosis in patients with non-alcoholic fatty liver disease. Ann Med. 2024;56(1):2409342.  188. Zhang F, Han Y, Wu YF, Bao ZW, Zheng GJ, Liu JH, et al. Association between triglyceride glucose-body mass index and the staging of non-alcoholic steatohepatitis and fibrosis in patients with non-alcoholic fatty liver disease. ANNALS OF MEDICINE. 2024;56(1).  189. Zhang S, Du T, Li M, Jia J, Lu H, Lin X, et al. Triglyceride glucose-body mass index is effective in identifying nonalcoholic fatty liver disease in nonobese subjects. Medicine (United States). 2017;96(22).  190. Zhang S, Du T, Li M, Jia J, Lu H, Lin X, et al. Triglyceride glucose-body mass index is effective in identifying nonalcoholic fatty liver disease in nonobese subjects. Medicine (United States). 2017;96(22).  191. Zhang S, Du T, Li M, Jia J, Lu H, Lin X, et al. Triglyceride glucose-body mass index is effective in identifying nonalcoholic fatty liver disease in nonobese subjects. Medicine (Baltimore). 2017;96(22):e7041.  192. Zhang SJ, Du TT, Li MN, Jia J, Lu HM, Lin X, et al. Triglyceride glucose-body mass index is effective in identifying nonalcoholic fatty liver disease in nonobese subjects. MEDICINE. 2017;96(22).  193. Zhao J, Fan H, Wang T, Yu B, Mao S, Wang X, et al. TyG index is positively associated with risk of CHD and coronary atherosclerosis severity among NAFLD patients. Cardiovascular Diabetology. 2022;21(1).  194. Zhao J, Fan H, Wang T, Yu B, Mao S, Wang X, et al. TyG index is positively associated with risk of CHD and coronary atherosclerosis severity among NAFLD patients. Cardiovascular Diabetology. 2022;21(1).  195. Zhao J, Fan H, Wang T, Yu B, Mao S, Wang X, et al. TyG index is positively associated with risk of CHD and coronary atherosclerosis severity among NAFLD patients. Cardiovasc Diabetol. 2022;21(1):123.  196. Zhao JQ, Fan HX, Wang T, Yu B, Mao SB, Wang X, et al. TyG index is positively associated with risk of CHD and coronary atherosclerosis severity among NAFLD patients. CARDIOVASCULAR DIABETOLOGY. 2022;21(1).  197. Zhong X, Huang D, Chen R, Yao L, Ma R, Yu Y, et al. Positive association between insulin resistance and fatty liver disease in psoriasis: evidence from a cross-sectional study. Frontiers in Immunology. 2024;15.  198. Zhong X, Huang D, Chen R, Yao L, Ma R, Yu Y, et al. Positive association between insulin resistance and fatty liver disease in psoriasis: evidence from a cross-sectional study. Frontiers in Immunology. 2024;15.  199. Zhong X, Huang D, Chen R, Yao L, Ma R, Yu Y, et al. Positive association between insulin resistance and fatty liver disease in psoriasis: evidence from a cross-sectional study. Front Immunol. 2024;15:1388967.  200. Zhong XY, Huang DW, Chen RF, Yao LL, Ma R, Yu YY, et al. Positive association between insulin resistance and fatty liver disease in psoriasis: evidence from a cross-sectional study. FRONTIERS IN IMMUNOLOGY. 2024;15.  201. Zou H, Ma X, Zhang F, Xie Y. Comparison of the diagnostic performance of twelve noninvasive scores of metabolic dysfunction-associated fatty liver disease. Lipids in Health and Disease. 2023;22(1).  202. Zou H, Ma X, Zhang F, Xie Y. Comparison of the diagnostic performance of twelve noninvasive scores of metabolic dysfunction-associated fatty liver disease. Lipids in Health and Disease. 2023;22(1).  203. Zou H, Ma X, Zhang F, Xie Y. Comparison of the diagnostic performance of twelve noninvasive scores of metabolic dysfunction-associated fatty liver disease. Lipids Health Dis. 2023;22(1):145.  204. Zou HX, Ma XP, Zhang F, Xie Y. Comparison of the diagnostic performance of twelve noninvasive scores of metabolic dysfunction-associated fatty liver disease. LIPIDS IN HEALTH AND DISEASE. 2023;22(1). |
