# Supplementary material for: Association of triglyceride glucose-body mass index (TyG-BMI) with metabolic dysfunction-associated steatotic liver disease: A systematic review and meta-analysis
Source: PLoS One. 2025 Aug 4;20(8):e0324483. doi: 10.1371/journal.pone.0324483 (PMC12321072; doi:10.1371/journal.pone.0324483)
Supplement: S3 Table — (DOCX) [file pone.0324483.s003.docx]

**Table S3.** Excluded studies after full text review

| **Author, year** | **Title** | **Reason for exclusion** |
| --- | --- | --- |
| V. Bullón-Vela, 2020 (1) | Association between triglyceride glucose-body mass index and risk factors linked to non-alcoholic liver disease in subjects with metabolic syndrome | Conference abstract |
| Q. Chen, 2024 (2) | Association between triglyceride-glucose related indices and mortality among individuals with non-alcoholic fatty liver disease or metabolic dysfunction-associated steatotic liver disease | Undesired outcome |
| S. A. Hosseini, 2024 (3) | Assessment of the appropriate cutoff points for anthropometric indices and their relationship with cardio-metabolic indices to predict the risk of metabolic associated fatty liver disease | Undesired measure of effect |
| Lim, 2019 (4) | Validation of fatty liver index in a healthy Korean population and its comparison with triglyceride glucose index and its related parameters | Conference abstract |
| Y. Qiao, 2025 (5) | Association between triglyceride-glucose (TyG) related indices and cardiovascular diseases and mortality among individuals with metabolic dysfunction-associated steatotic liver disease: a cohort study of UK Biobank | Undesired outcome |
| Y. Xing, 2022 (6) | Associations Between GGT/HDL and MAFLD: A Cross-Sectional Study | Undesired measure of effect |
| F. Zhang, 2024 (7) | Association between triglyceride glucose-body mass index and the staging of non-alcoholic steatohepatitis and fibrosis in patients with non-alcoholic fatty liver disease | Undesired outcome |
| X. Zhong, 2024 (8) | Positive association between insulin resistance and fatty liver disease in psoriasis: evidence from a cross-sectional study | Undesired measure of effect |

References:

1. Bullón-Vela V, Abete I, Zulet MA, Martínez JA. Association between triglyceride glucose-body mass index and risk factors linked to non-alcoholic liver disease in subjects with metabolic syndrome. Proceedings of the Nutrition Society. 2020;79(OCE2).

2. Chen Q, Hu P, Hou X, Sun Y, Jiao M, Peng L, et al. Association between triglyceride-glucose related indices and mortality among individuals with non-alcoholic fatty liver disease or metabolic dysfunction-associated steatotic liver disease. Cardiovascular Diabetology. 2024;23(1).

3. Hosseini SA, Alipour M, Sarvandian S, Haghighat N, Bazyar H, Aghakhani L. Assessment of the appropriate cutoff points for anthropometric indices and their relationship with cardio-metabolic indices to predict the risk of metabolic associated fatty liver disease. BMC Endocrine Disorders. 2024;24(1).

4. Lim J. Validation of fatty liver index in a healthy Korean population and its comparison with triglyceride glucose index and its related parameters. Clinica Chimica Acta. 2019;493:S374.

5. Qiao Y, Wang Y, Chen C, Huang Y, Zhao C. Association between triglyceride-glucose (TyG) related indices and cardiovascular diseases and mortality among individuals with metabolic dysfunction-associated steatotic liver disease: a cohort study of UK Biobank. Cardiovasc Diabetol. 2025;24(1):12.

6. Xing Y, Chen J, Liu J, Ma H. Associations Between GGT/HDL and MAFLD: A Cross-Sectional Study. Diabetes, Metabolic Syndrome and Obesity. 2022;15:383-94.

7. Zhang F, Han Y, Wu Y, Bao Z, Zheng G, Liu J, et al. Association between triglyceride glucose-body mass index and the staging of non-alcoholic steatohepatitis and fibrosis in patients with non-alcoholic fatty liver disease. Annals of Medicine. 2024;56(1).

8. Zhong X, Huang D, Chen R, Yao L, Ma R, Yu Y, et al. Positive association between insulin resistance and fatty liver disease in psoriasis: evidence from a cross-sectional study. Frontiers in Immunology. 2024;15.
