# Supplementary figures and images for: Association of triglyceride glucose-body mass index (TyG-BMI) with metabolic dysfunction-associated steatotic liver disease: A systematic review and meta-analysis
Source: PLoS One. 2025 Aug 4;20(8):e0324483. doi: 10.1371/journal.pone.0324483 (PMC12321072; doi:10.1371/journal.pone.0324483)

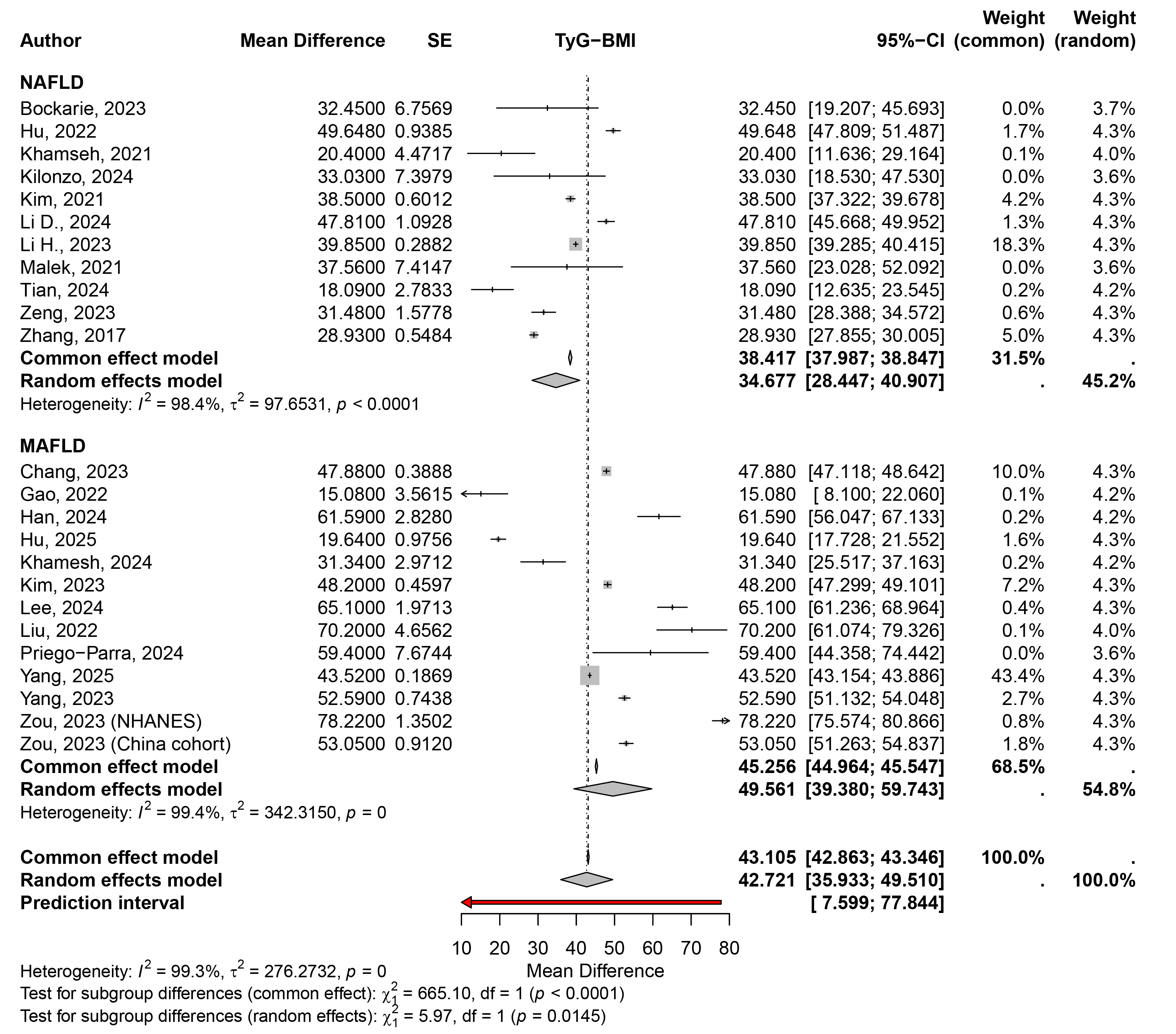

Supplement: S1 Fig — (TIF) [file pone.0324483.s005.tif]

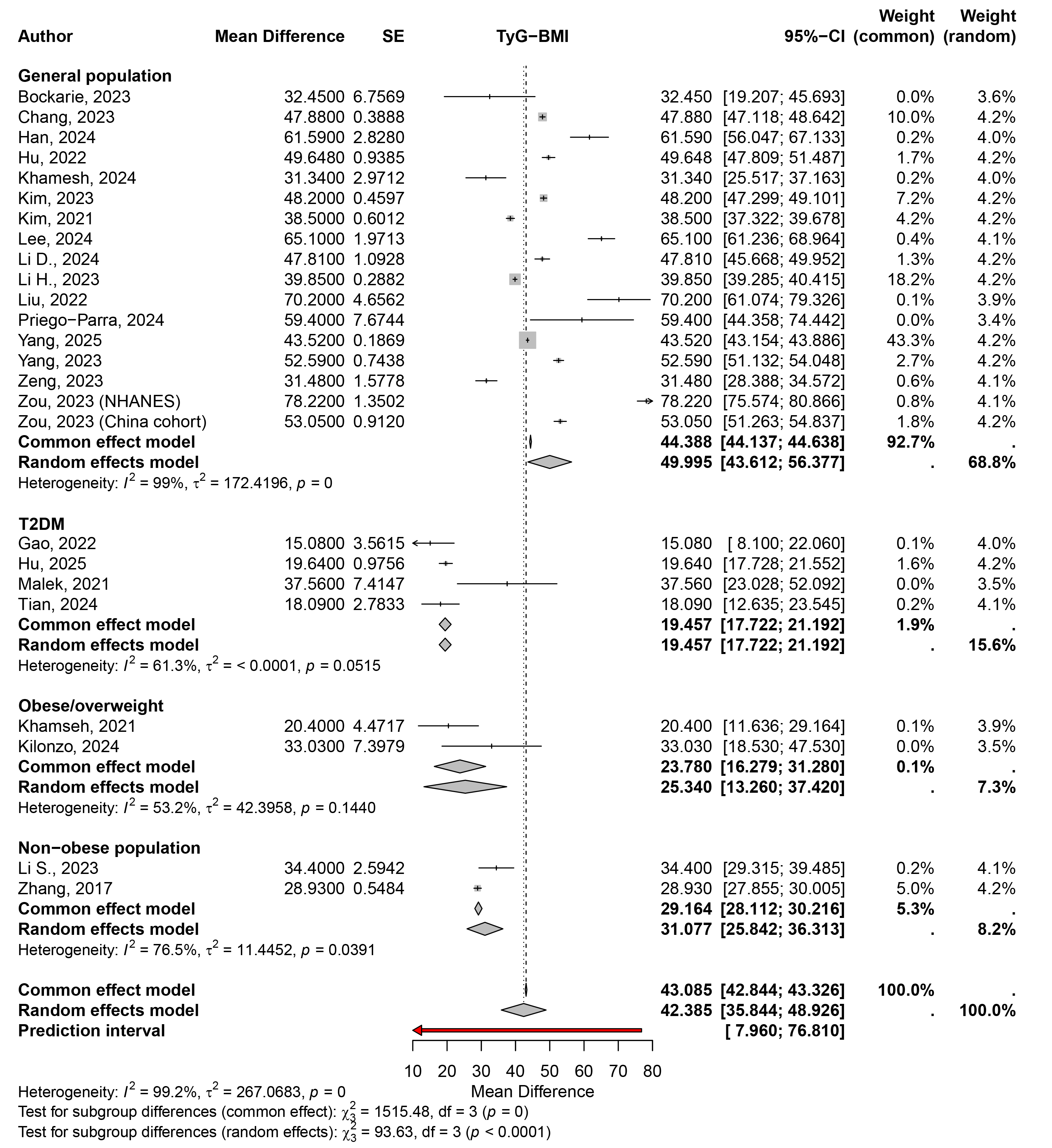

Supplement: S2 Fig — (TIF) [file pone.0324483.s006.tif]

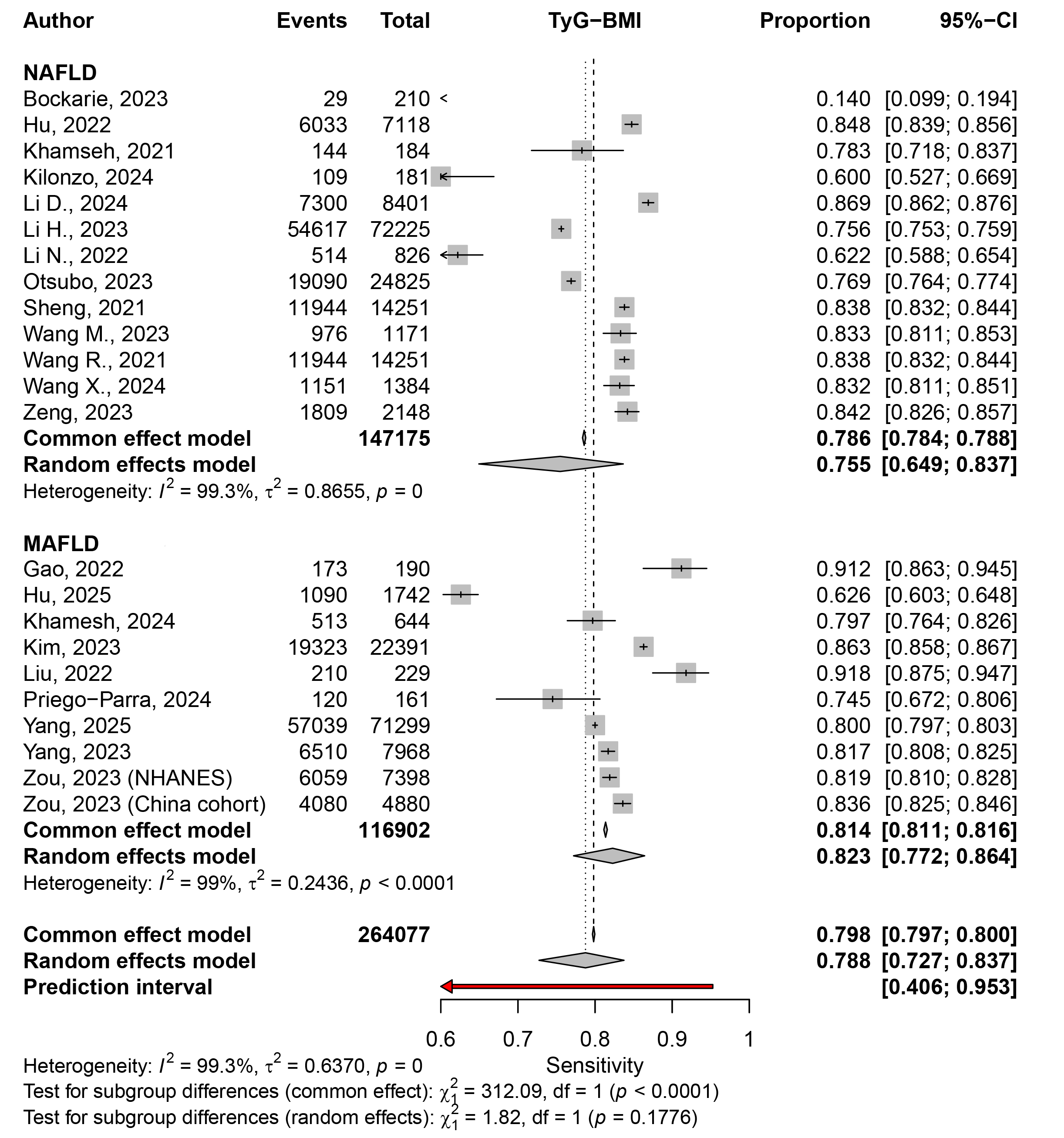

Supplement: S3 Fig — (TIF) [file pone.0324483.s007.tif]

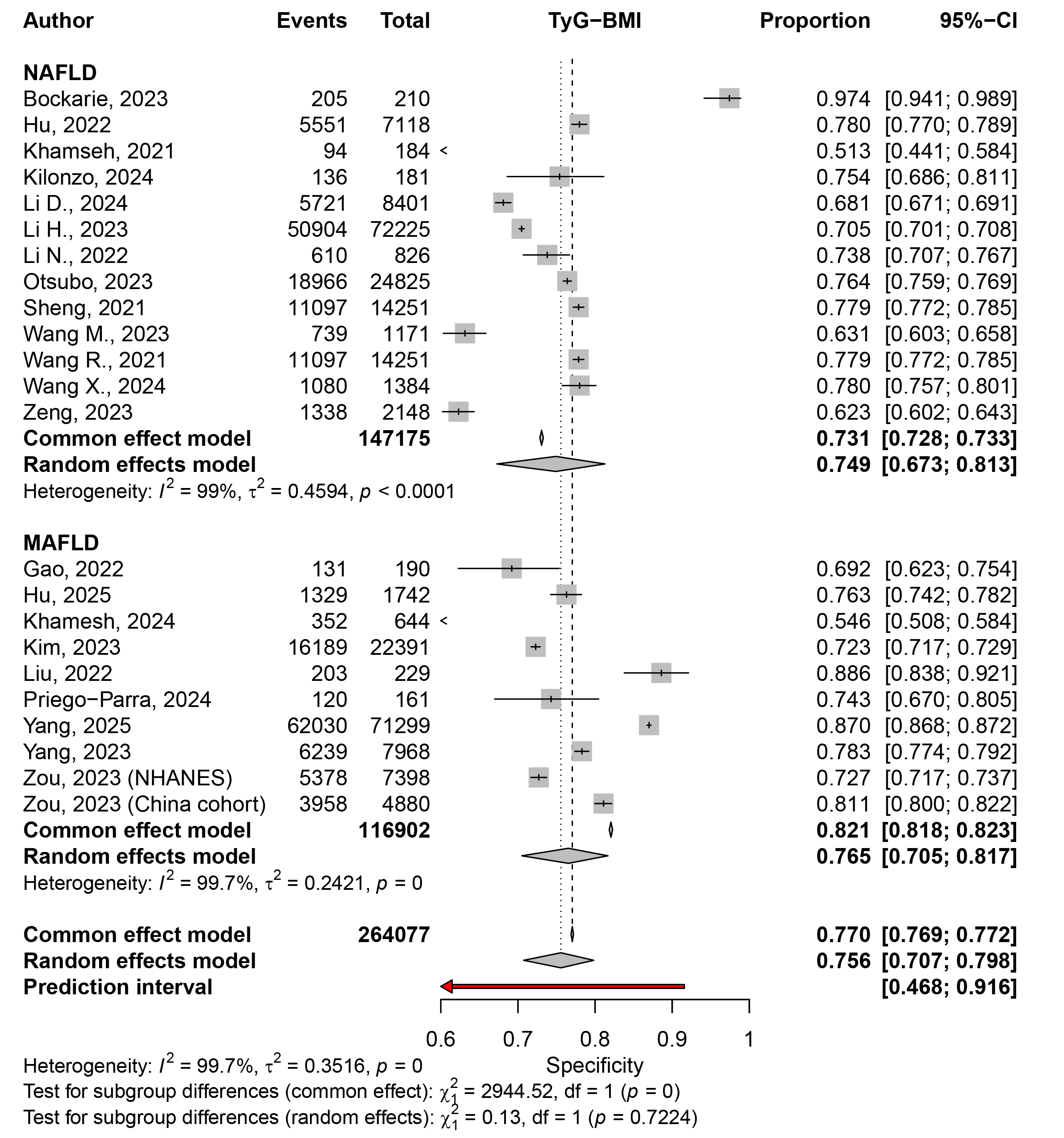

Supplement: S4 Fig — (TIF) [file pone.0324483.s008.tif]

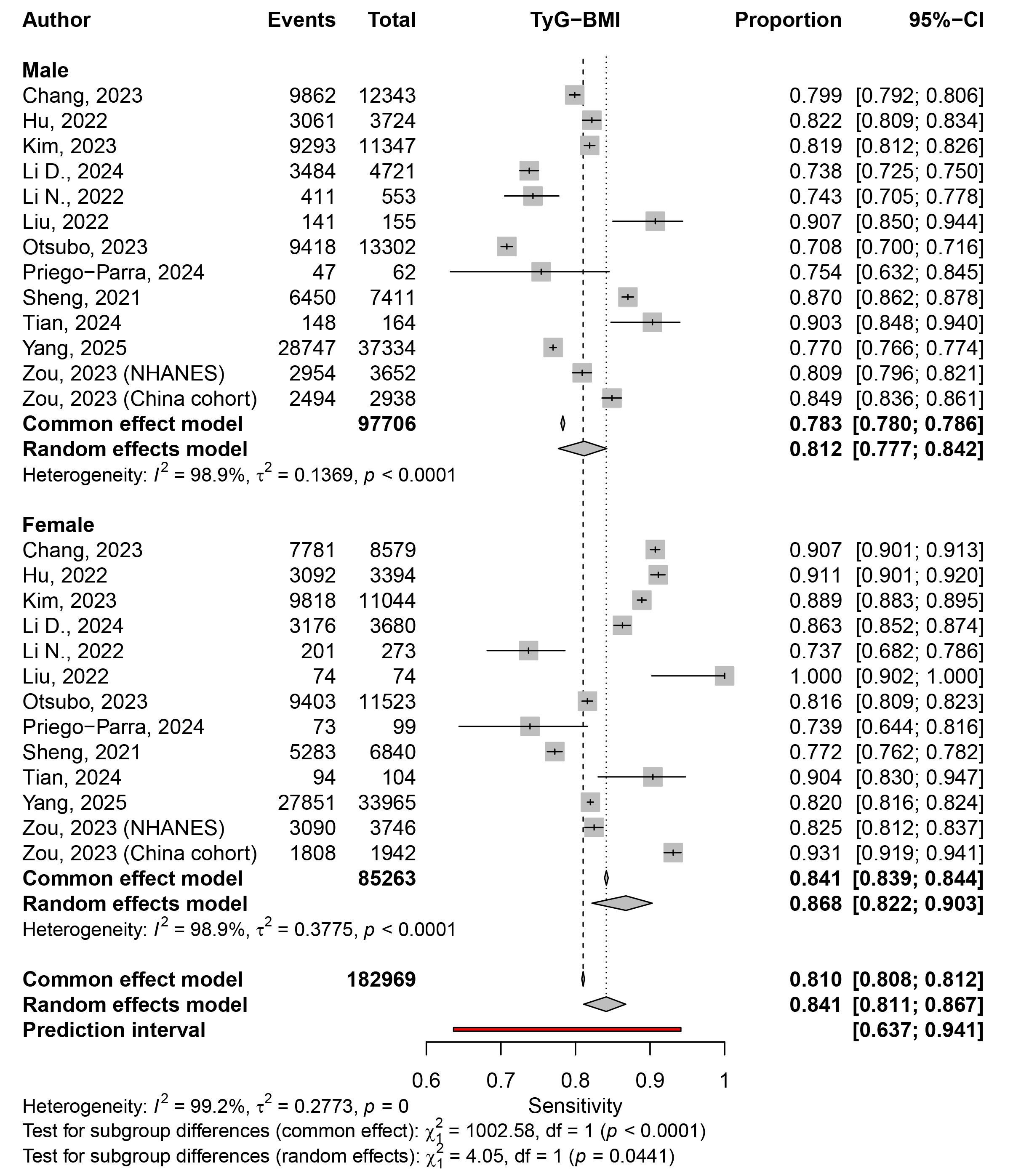

Supplement: S5 Fig — (TIF) [file pone.0324483.s009.tif]

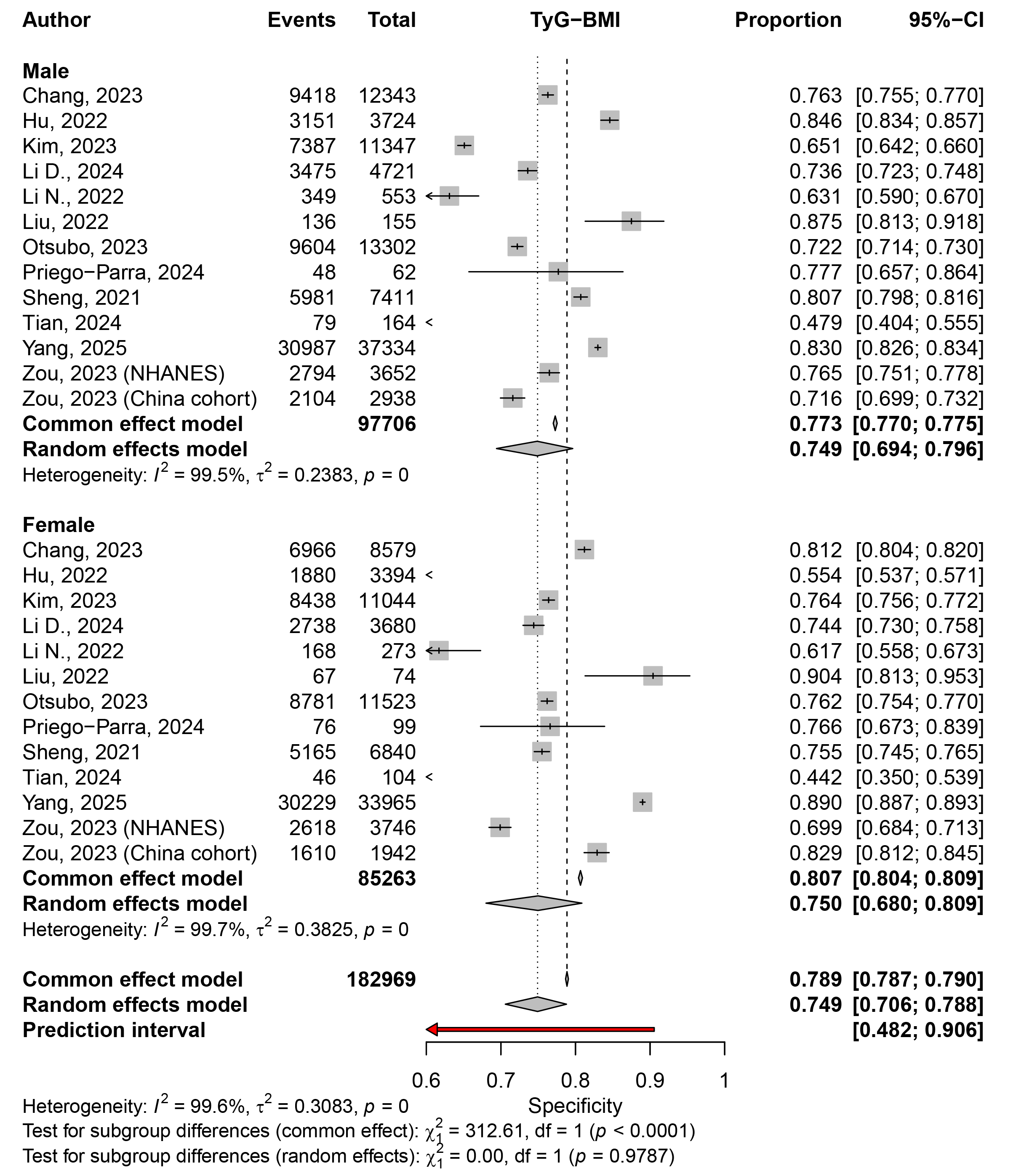

Supplement: S6 Fig — (TIF) [file pone.0324483.s010.tif]

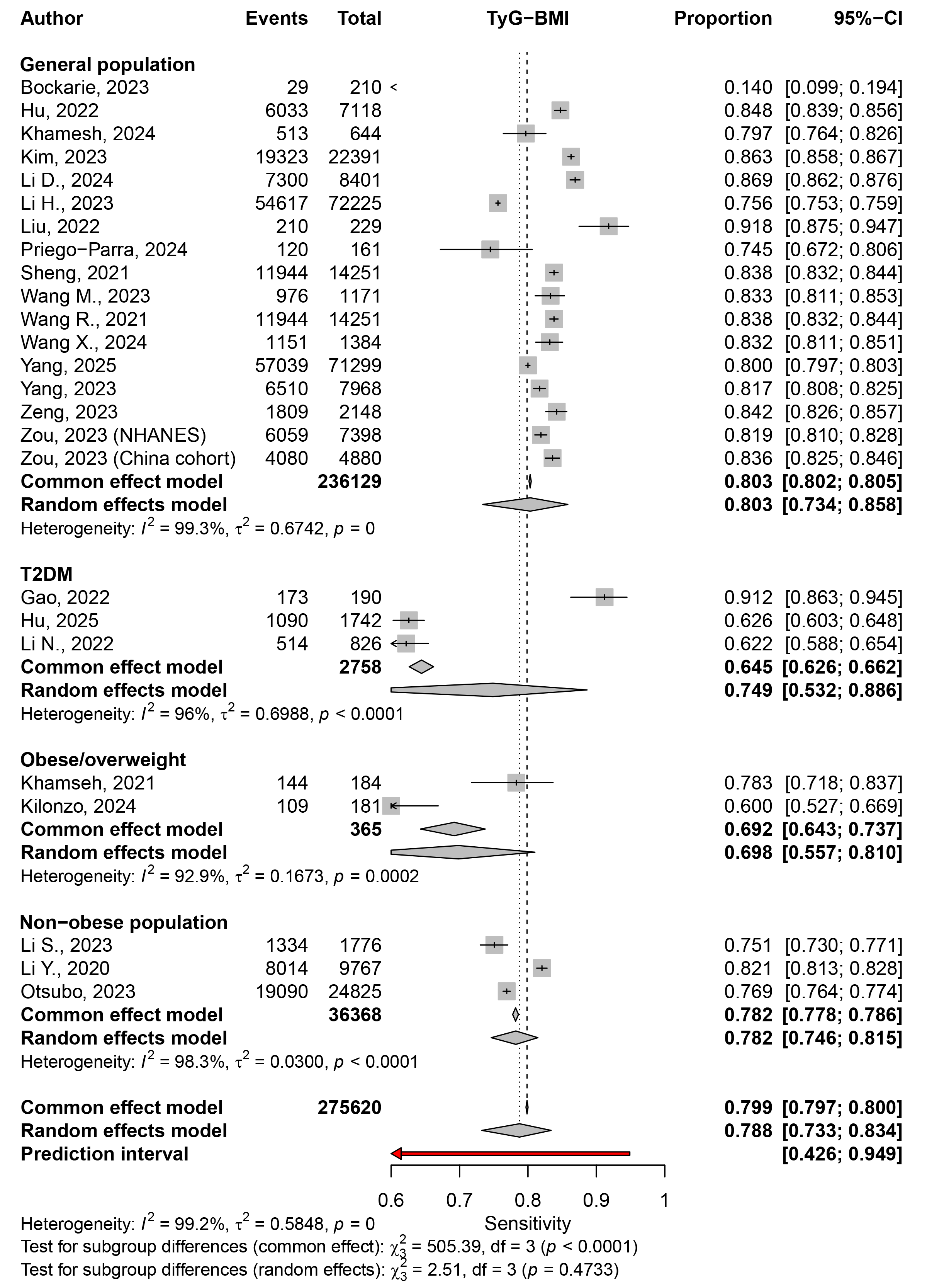

Supplement: S7 Fig — (TIF) [file pone.0324483.s011.tif]

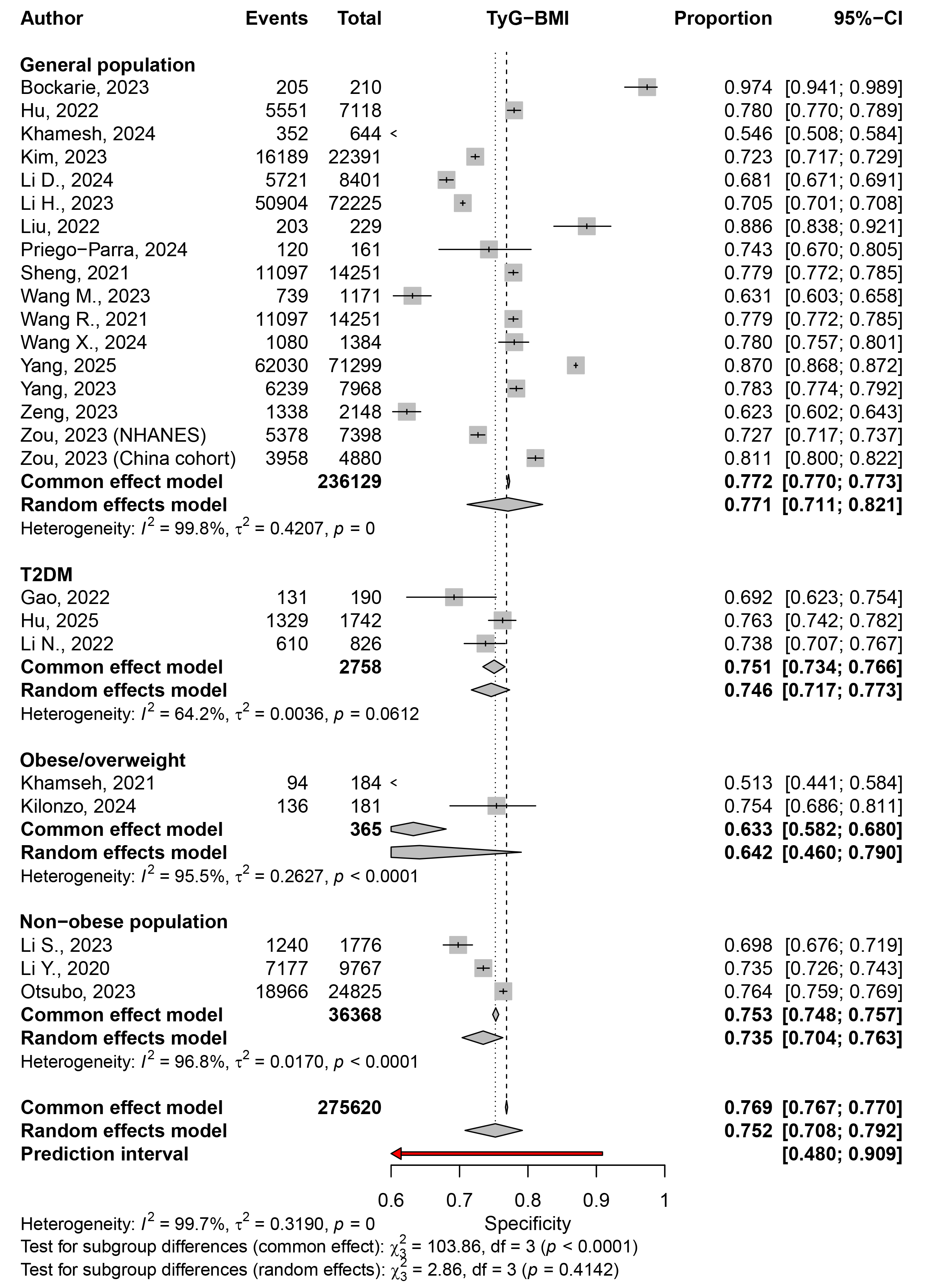

Supplement: S8 Fig — (TIF) [file pone.0324483.s012.tif]

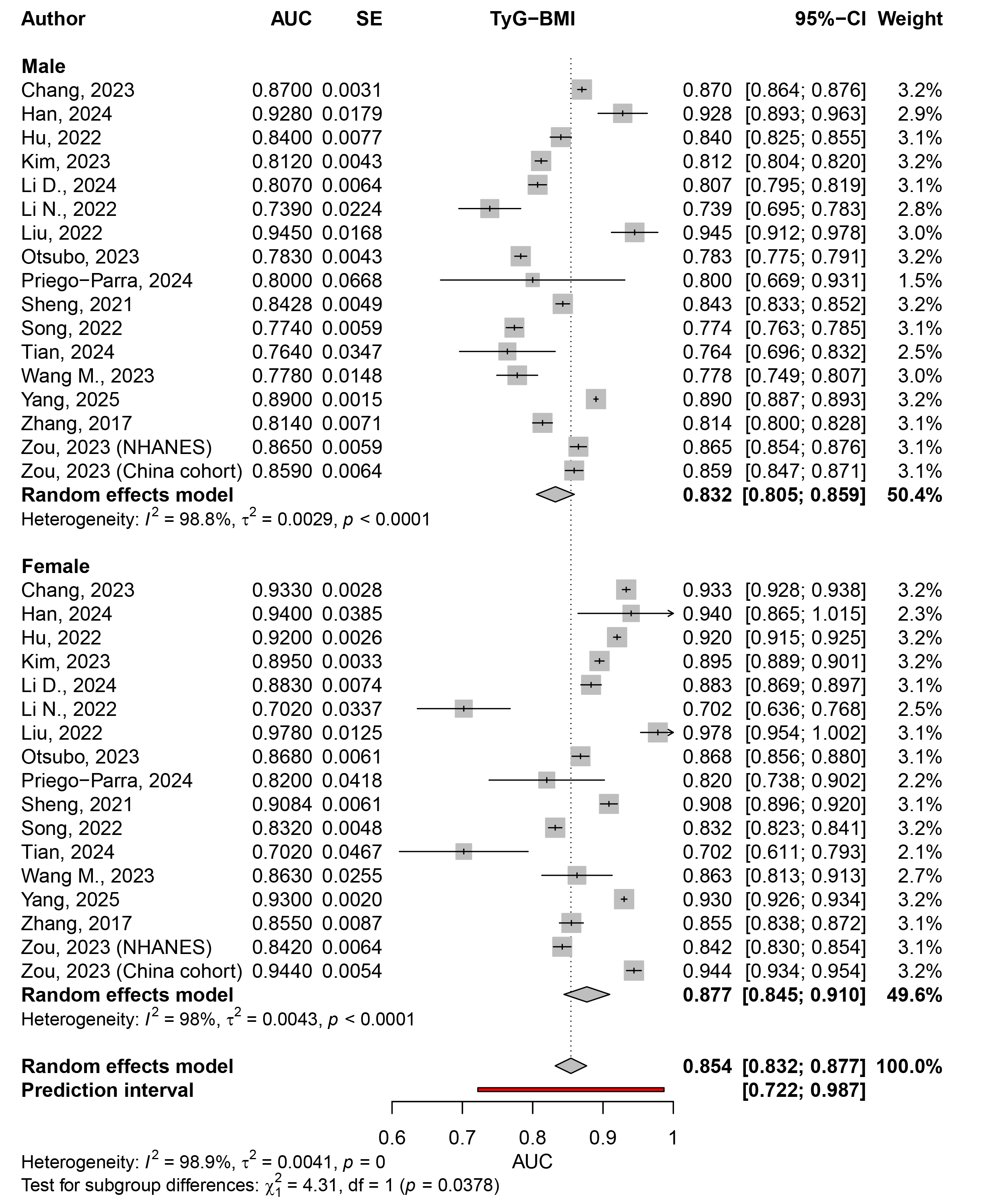

Supplement: S9 Fig — (TIF) [file pone.0324483.s013.tif]

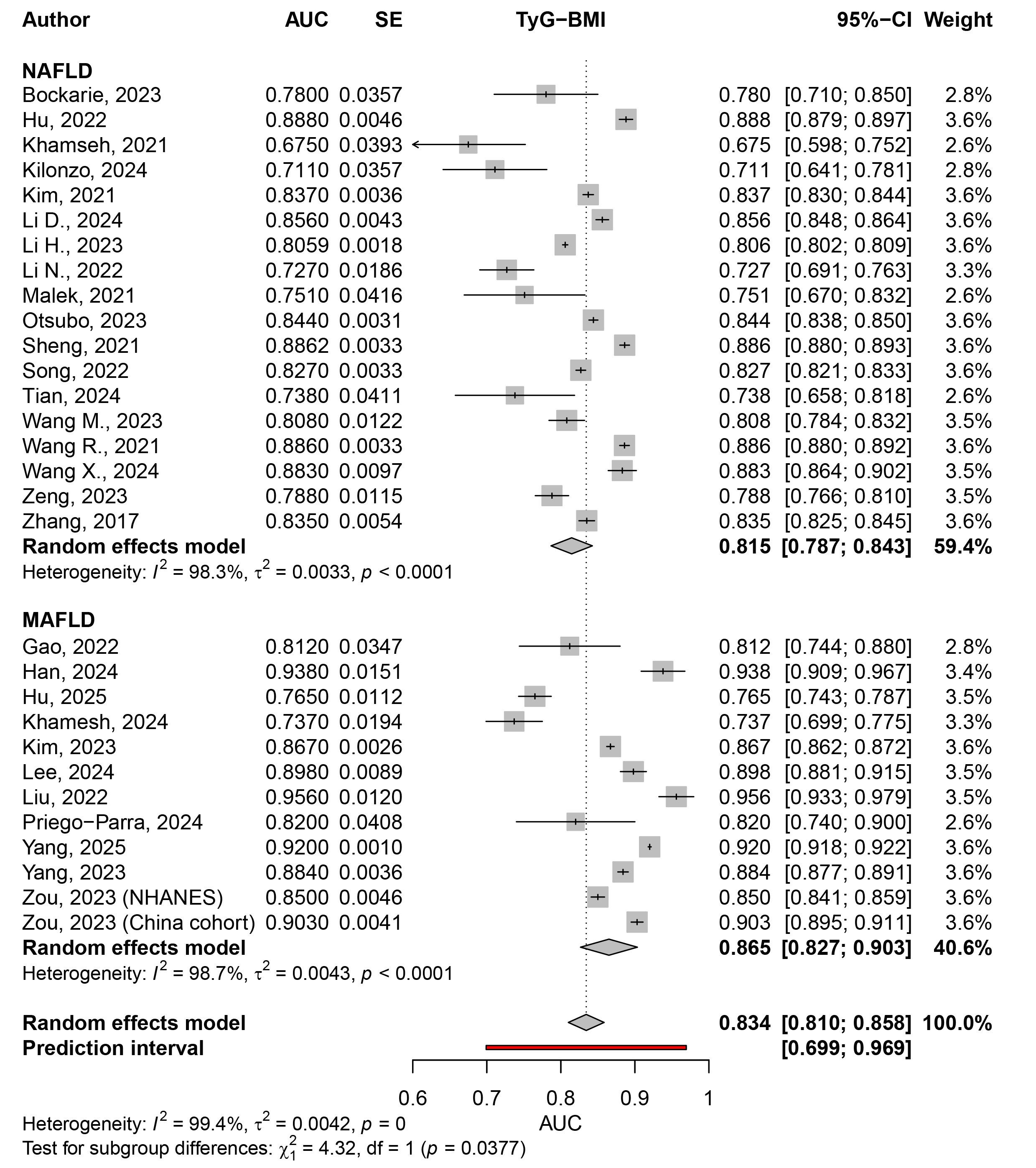

Supplement: S10 Fig — (TIF) [file pone.0324483.s014.tif]

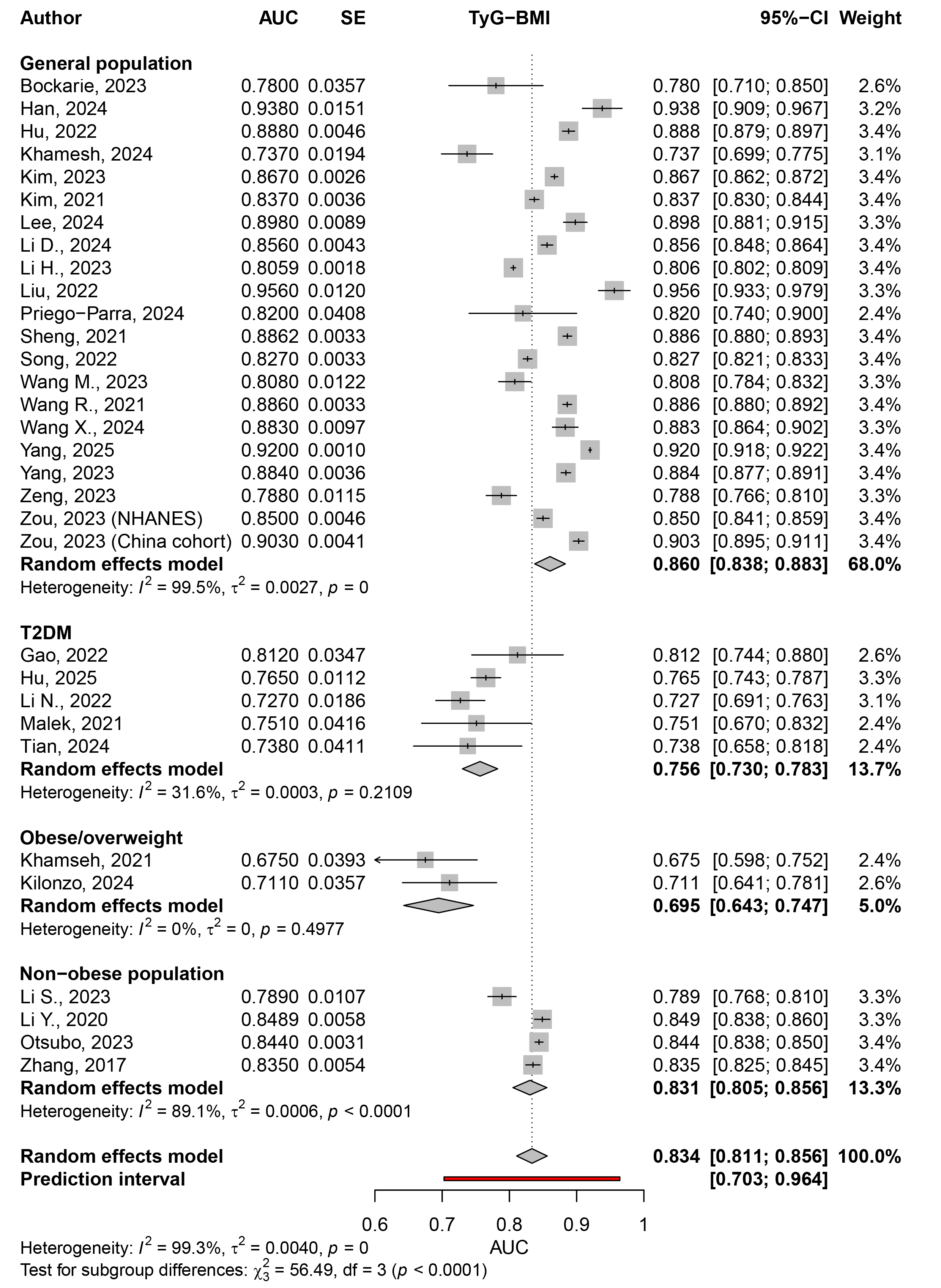

Supplement: S11 Fig — (TIF) [file pone.0324483.s015.tif]
